# Genome-wide analysis of wheat xyloglucan endotransglucosylase/hydrolase (XTH) gene family revealed *TaXTH17* involved in abiotic stress responses

**DOI:** 10.1186/s12870-024-05370-4

**Published:** 2024-07-06

**Authors:** Huihui Bi, Zeliang Liu, Shanshan Liu, Wenchen Qiao, Kunpu Zhang, Minghui Zhao, Daowen Wang

**Affiliations:** 1 National Key Laboratory of Wheat and Maize Crop Science, College of Agronomy, Henan Agricultural University, Zhengzhou, 450002 China; 2 Key Laboratory of Crop Drought Resistance Research of Hebei Province, Dry Farming Institute, Hebei Academy of Agriculture and Forestry Sciences, Hengshui, 053000 China

**Keywords:** *Triticum aestivum*, Genome-wide, Environmental stresses, Transgenic, Salt, Drought, Functional analysis

## Abstract

**Background:**

Environmental stresses, including high salinity and drought, severely diminish wheat yield and quality globally. The xyloglucan endotransglucosylase/hydrolase (XTH) family represents a class of cell wall-modifying enzymes and plays important roles in plants growth, development and stress adaptation. However, systematic analyses of XTH family genes and their functions under salt and drought stresses have not been undertaken in wheat.

**Results:**

In this study, we identified a total of 135 *XTH* genes in wheat, which were clustered into three evolutionary groups. These *TaXTHs* were unevenly distributed on 21 chromosomes of wheat with a majority of *TaXTHs* located on homelogous groups 2, 3 and 7. Gene duplication analysis revealed that segmental and tandem duplication were the main reasons for the expansion of *XTH* family in wheat. Interaction network predictions indicated that TaXTHs could interact with multiple proteins, including three kinases, one methyltransferase and one gibberellin-regulated protein. The promoters of the *TaXTH* genes harbored various cis-acting elements related to stress and hormone responses. RNA-seq data analyses showed that some *TaXTH* genes were induced by salt and drought stresses. Furthermore, we verified that *TaXTH17* was induced by abiotic stresses and phytohormone treatments, and demonstrated that TaXTH17 was localized in the secretory pathway and cell wall. Functional analyses conducted in heterologous expression systems and in wheat established that *TaXTH17* plays a negative role in plant resistance to salt and drought.

**Conclusions:**

We identified 135 *XTH* genes in wheat and conducted comprehensive analyses of their phylogenetic relationships, gene structures, conserved motifs, gene duplication events, chromosome locations, interaction networks, cis-acting elements and gene expression patterns. Furthermore, we provided solid evidence supporting the notion that *TaXTH17* plays a negative role in plant resistance to salt and drought stresses. Collectively, our results provide valuable insights into understanding wheat *XTHs*, particularly their involvement in plant stress responses, and establish a foundation for further functional and mechanistic studies of *TaXTHs*.

**Supplementary Information:**

The online version contains supplementary material available at 10.1186/s12870-024-05370-4.

## Background

As a crucial component outside the cell membrane, the plant cell wall is a flexible structure that can be promptly remodeled in response to developmental or environmental cues. It primarily comprises polysaccharide polymers, including cellulose, hemicellulose, pectin and glycoproteins. The plant cell wall plays multiple vital roles, including shaping plant morphology and construction, providing mechanical support for cells, and protecting against biotic and abiotic stresses [1–3]. In recent years, accumulating studies have shown that the loosening and rearrangement of the cell wall are critical events in processes such as cell number increase, volume expansion, shape alteration, and stress adaptation [4]. In particular, cell wall modifying proteins and their structural remodeling are crucial for plants’ responses and adaptation to changing environmental conditions, including drought [4–6].

One of the factors responsible for the plasticity of the cell wall is the xyloglucan endotransglucosylase/hydrolase (XTH) family, which cleaves and rejoins xyloglucan molecules. Enzymes belonging to the XTH family, along with members of the glycoside hydrolase family 16 (GH16), are believed to influence cell wall mechanics and expansion by cleaving and reconnecting xyloglucan [7–9]. The XTH family possesses two catalytic functions: xyloglucan endotransglucosylase (XET) activity and xyloglucan endohydrolase (XEH) activity, the latter specifically hydrolyzing glycosidic bonds of xyloglucan to promote cell wall expansion and morphogenesis [7]. XTH proteins are generally classified into four groups based on their structural characteristics, namely groups I/II, III A, III B and the ancestral group [10, 11]. Among the XTHs reported to date, those exhibiting glycosyltransferase activity are predominantly in Group I/II, while those with hydrolase activity are mainly in Group III. GH16 family members generally contain Glyco_hydro_16 conserved structural motif and N-glycosylation site [12]. With the rapid advancement of plant genome sequencing, the identification of XTH family members and the regulation of gene expression have been extensively studied across various plant species, including *Arabidopsis thaliana* [10], *Oryza sativa* [13], *Solanum lycopersicum* [14], *Glycine max* [15], *Hordeum vulgare* [16], two Brassica species, *Brassica rapa* and *Brassica oleracea* [17], as well as *Camellia sinensis* [18].

Numerous studies have demonstrated that XTHs play important roles in plant responses to various environmental stresses. For instance, overexpression of *CaXTH3* in tomato and Arabidopsis improved salt and drought tolerance of transgenic plants by influencing stomatal closure [19, 20]. The constitutive expression of *PeXTH* in tobacco increased resistance to salt and cadmium [12, 21]. Loss-of-function mutations in Arabidopsis *xth15*, *xth17* and *xth31* exhibited enhanced aluminum tolerance compared with wild type controls [22–24]. *AtXTH30* negatively affects salt tolerance in Arabidopsis via modulating xyloglucan side chains, altering the abundance of xyloglucan-derived oligosaccharide, cellulose synthesis, and cortical microtubule stability [25]. *XTH19* and *XTH23* are involved in lateral root development in Arabidopsis via the BES1-dependent pathway, and contribute to the adaptation of lateral roots to salt stress [26]. In another study, compared to the wild type control, the *xth19* mutant showed decreased freezing tolerance after cold and sub-zero acclimation, which was resulted from alterations in cell wall composition and structure [27]. In *Brachypodium distachyon*, a model species of monocots, higher expression levels of *XTH* genes and genes involved in xylan biosynthesis are closely associated with drought tolerance [6].

Wheat (*Triticum aestivum* L.) stands as one of the most important staple food crops globally. However, environmental stresses such as drought and high salinity significantly diminish wheat yield and quality worldwide. It is generally believed that it is an economical and effective way to improve the stress resistance of wheat by exploiting and utilizing elite stress resistance genes. In a previous study, it was estimated that there are at least 57 XTH members in the common wheat genomes based on the wheat EST database [28]. The significant roles of XTHs in plant adaptation to environmental stresses as well as the completion of the reference genome sequencing of hexaploid wheat prompted us to conduct a comprehensive genome-wide analysis of wheat *XTH* genes with the aims to identify *TaXTHs* involved in abiotic stress responses. In this study, we identified 135 wheat XTH family genes using publicly available wheat genome data. Subsequently, we analyzed the phylogenetic relationships, gene structures, conserved motifs, gene duplication, chromosome locations, interaction networks, cis-acting elements and gene expression patterns of these *XTHs*. Subcellular localization analyses revealed that TaXTH17-1D, a member of the wheat XTH family, is localized in the secretory pathway and cell wall. Heterologous expression in yeast and Arabidopsis demonstrated that *TaXTH17-1D* negatively affects plant tolerance to salt and drought stresses, while silencing *TaXTH17* in wheat enhances drought tolerance. This study lays a foundation for further elucidation of XTH gene functions and molecular mechanisms, and sheds light on the potential of *TaXTH17* in abiotic stress tolerance improvement.

## Results

### Genome-wide identification of *XTHs* in wheat

**Fig. 1:**
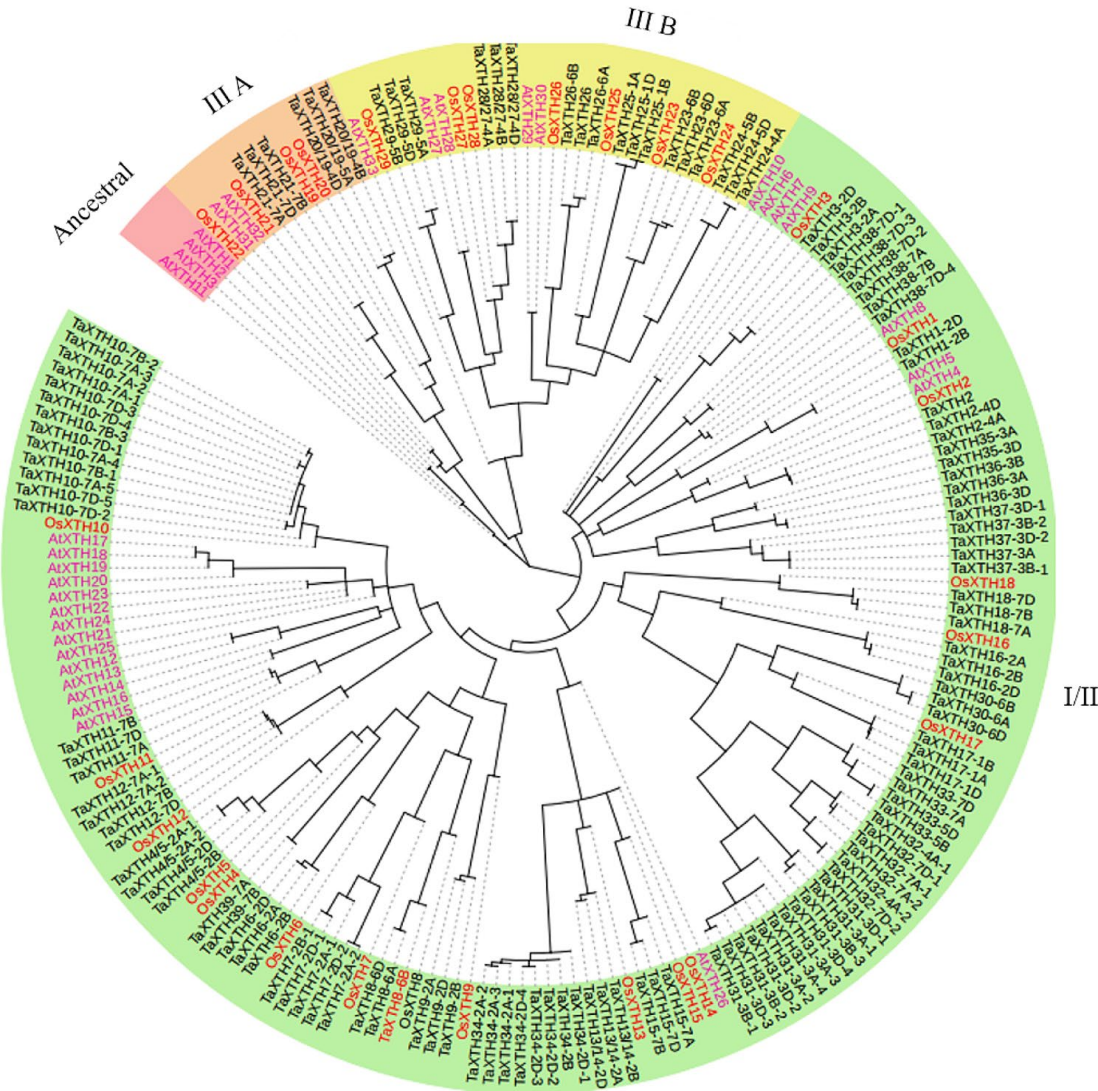
Phylogenetic analysis of XTH gene family in wheat, rice and Arabidopsis. The XTH family proteins were divided into three clusters (I/II, III A and III B), which were indicated with different colors. Ta, *Triticum aestivum*; Os, *Oryza sativa*; At, *Arabidopsis thaliana*

Using the method described below, we identified a total of 135 *TaXTH* genes from wheat, encoding 39 family members. To understand the evolutionary relationships of XTH gene family members, we constructed a phylogenetic tree using the full-length protein sequences of 135 wheat TaXTHs, 29 rice OsXTHs and 33 Arabidopsis AtXTHs (Fig. 1). Based on the classification standard of Arabidopsis and rice XTHs, TaXTHs were divided into three evolutionary groups, namely group I/II, group III A and group III B, which contain 111, 6 and 18 TaXTH proteins, respectively. The numbers of XTH proteins in wheat, rice and Arabidopsis are the highest in Group I/II, accounting for 82%, 62% and 67% of TaXTHs, OsXTHs and AtXTHs proteins, respectively. TaXTHs were named according to their homologous relationships with rice XTH genes. Since there are 29 XTHs in rice, the TaXTHs without rice homologs were named from TaXTH30 to TaXTH39. In addition, the chromosome position was incorporated into gene names, denoted by positions like 2B, indicating it is located on the 2B chromosome. Notably, some genes have multiple sequences on the same chromosome, thus in such cases numbers 1, 2, etc. were added after chromosome positions. Basic information about TaXTHs is presented in Table S1. The proteins encoded by *TaXTHs* range from 209 to 363 amino acids in length, with TaXTH25-1 A being the longest. Their molecular weights vary from a maximum of 39.92 kDa to a minimum of 23.72 kDa. Most TaXTHs contain signal peptide sequences with several exceptions. All TaXTHs were predicted to localize in the cell wall, with a few also localized in the cytoplasm.

### Duplication and chromosomal location analyses of *TaXTHs*

**Fig. 2:**
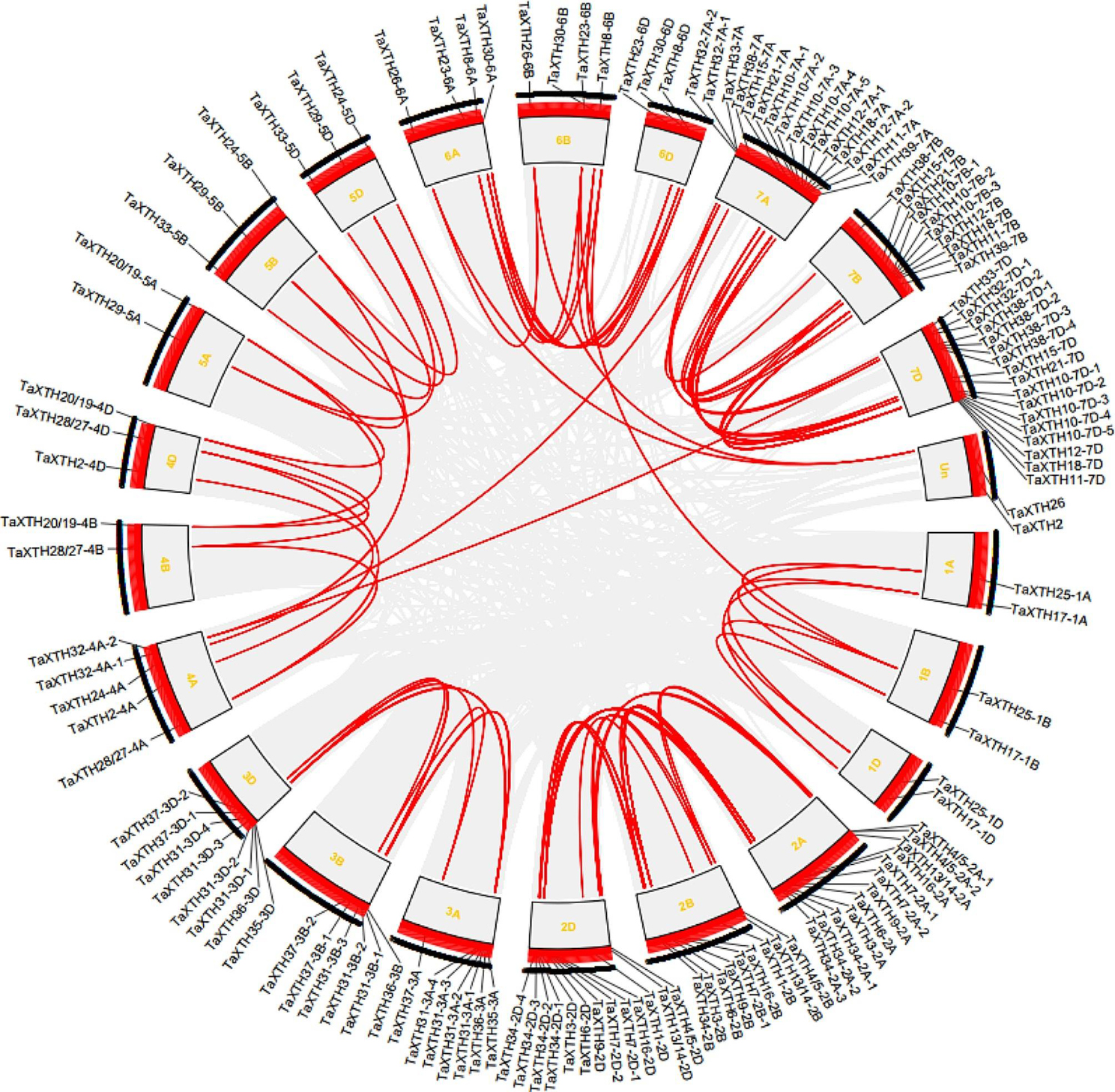
Gene duplication and chromosomal distribution of wheat *XTH* genes. The red lines indicate segmental duplicated *XTH* gene pairs. Chromosome numbers in yellow color are displayed next to each chromosome. Un, location unknown genes

In plants, the majority of genes exist as members of gene families, with segmental duplication and tandem duplication being two main forces leading to gene family expansion. Our investigation uncovered 88 pairs of segmentally duplicated *TaXTH* genes, constituting 68.89% of *TaXTH* genes (Fig. 2). Notably, segmental duplication primarily occurred between homelogous genes. In addition, there are 66 pairs of tandemly duplicated genes, defined as genes located within 200 kb, accounting for 48.89% of *TaXTH* genes. These tandemly duplicated genes are mainly distributed on chromosomes 2A, 2D, 3D, 7A, 7D, and include *TaXTH10-7D*, *TaXTH31-3A*, *TaXTH31-3B*, *TaXTH31-3D*, *TaXTH34-2A*, *TaXTH34-2D* gene clusters, etc.

Besides, from Fig. 2 we observed that among the 135 identified *TaXTH* genes, 133 are unevenly distributed across the 21 chromosomes of wheat, while the chromosome locations of 2 *TaXTHs* remain unknown. The majority of *TaXTHs* are situated on homelogous groups 2, 3 and 7. Chromosome 7D harbors the highest number of genes, with 17, followed by chromosome 7 A with 16 genes. Conversely, chromosomes 1A, 1B, 1D, 4B, and 5A host the fewest genes, each containing only two. At the subgenome level, 48 *TaXTHs* are located on genome A, 36 on genome B, and 49 on genome D.

### Gene structure and conserved motif analyses of *TaXTHs*

Gene structure analyses revealed that *TaXTHs* contain similar numbers of exons and introns (Figure S1). All *TaXTHs* in group III A consist of three exons; except for *TaXTH24*, which has only one exon, while *TaXTHs* in group III B contain 2–4 exons. In group I/II, there are 62 genes with 3 exons, 33 genes with 2 exons, and 16 genes with 4 exons. In terms of motif constitution, most TaXTHs have all 10 motifs, with motif 2, motif 3 and motif 8 being highly conserved and present in all TaXTHs. TaXTHs in group III A and group III B contain all 10 motifs except for TaXTH21-7B in group III A and TaXTH29 in group III B. The number of motifs in group I/II varies from 7 to 10, with more than 20 TaXTHs possessing 9 motifs and a few TaXTHs containing 8 or 7 motifs.

### Interaction networks analysis of TaXTHs

**Fig. 3:**
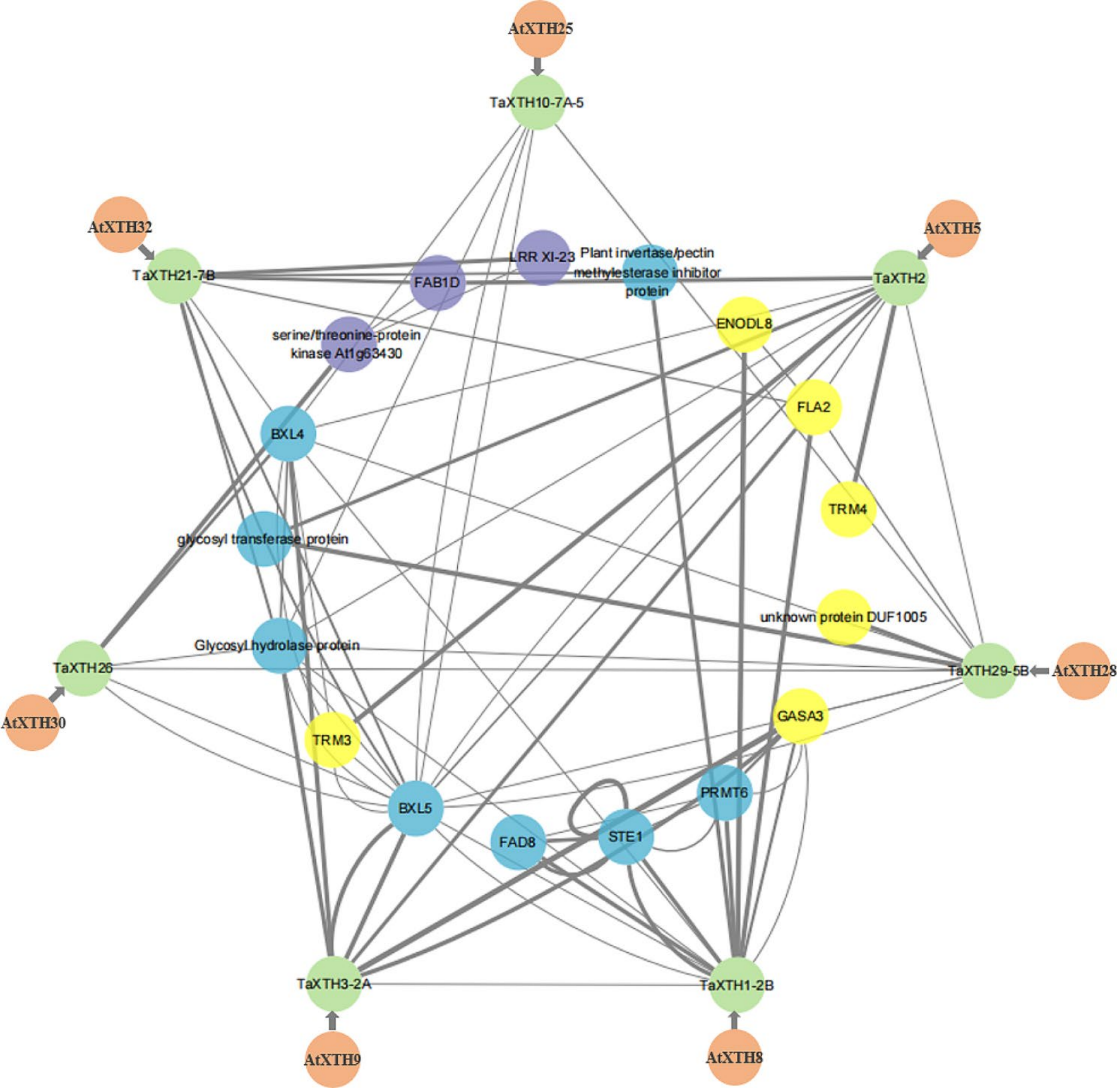
Predicted protein-protein interaction networks of TaXTHs. TaXTH proteins are represented by green circles, and their homologs in Arabidopsis are denoted by orange circles. The proteins that interact with TaXTHs are indicated by purple, blue and yellow circles, which represent kinases, other types of enzymes and nonenzyme proteins, respectively. Interacted proteins are linked by gray lines, and thicker lines indicate greater possibility of interaction

To gain insights into the biological function and regulatory network of TaXTHs, the interaction network of XTH family proteins in wheat was analyzed. As a result, seven TaXTHs were found to have homologous relationships with counterparts in Arabidopsis, and 17 interacting proteins were identified (Fig. 3). Except for three unknown proteins, the interacting proteins can be categorized into three groups: three kinases, namely leucine-rich receptor-like protein kinase family protein LRR XI-23, probable LRR receptor-like serine/threonine protein kinase At1g63430, and putative 1-phosphatidylinositol-3-phosphate 5-kinase FAB1D; eight other types of enzymes, including plant invertase/pectin methylesterase inhibitor superfamily protein, probable protein arginine N-methyltransferase 6 (PRMT6), glycosyltransferase protein, glycosyl hydrolase protein, beta-D-xylosidase 4 (BXL4), probable beta-D-xylosidase 5 (BXL5), temperature-sensitive omega-3 fatty acid desaturase (FAD8), and delta(7)-sterol-C5(6)-desaturase 1 (STE1); and three nonenzymes, namely gibberellin-regulated protein 3 (GASA3), fasciclin-like arabinogalactan protein 2 (FLA2), and early nodulin-like protein 8 (ENODL8).

### Cis-acting elements analysis of *TaXTHs*

Cis-acting elements in gene promoter regions are directly associated with gene transcriptional regulation. To understand the expression of XTH family genes in response to abiotic stresses and hormone induction, we analyzed the cis-acting elements present in *TaXTHs*. As shown in Table S2, the promoter regions of *TaXTHs* contain various hormone-related cis-elements, including abscisic acid response element (ABRE), methyl jasmonate response element (CGTCA-motif/TGACG-motif), auxin response element (Aux-core), gibberellin response element (GARE-motif), and salicylic acid response element (TCA-element). The numbers of *TaXTHs* containing the above cis-elements were found to be 126, 112, 71, 61 and 34, respectively. As for stress response elements, 98 *TaXTHs* contain the MYB binding site involved in drought-induced stress (MBS), 96 genes contain anaerobic response elements (ARE), 56 genes contain low temperature response elements (LTR), 42 genes contain hypoxia response elements (GC-motif), 18 genes contain defense response elements (TC-rich repeats) and 5 genes contain wounding response elements (WUN-motif). In addition, it was found that most *TaXTHs* contained 7–19 stress and hormone-related cis-acting elements.

### Expression profiles of *TaXTHs*

**Fig. 4:**
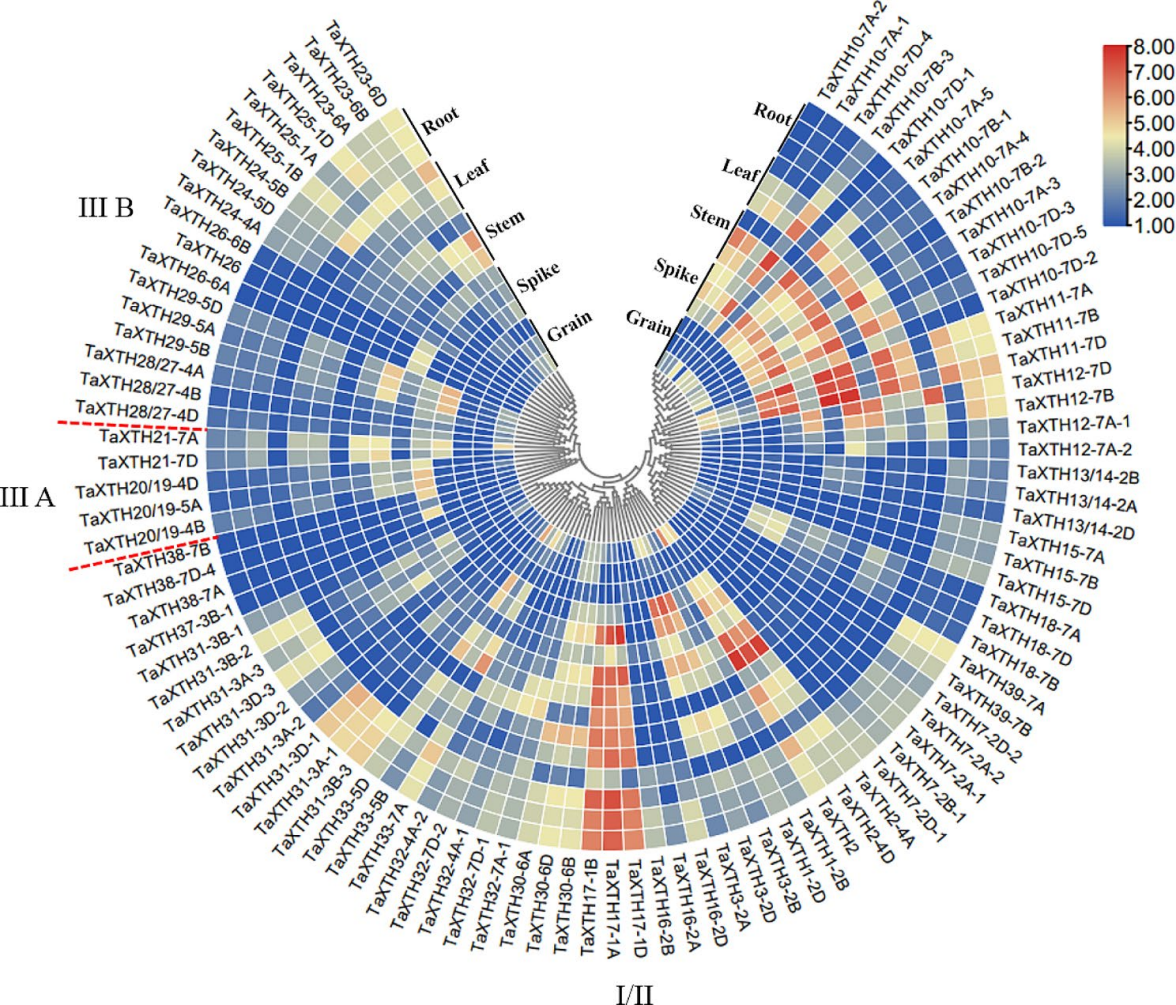
Circled heat map showing the spatio-temporal expression patterns of wheat *TaXTH* genes in wheat. RNA-Seq data of bread wheat cultivar Chinese spring were retrieved from WheatExp database. Data include expression levels in five different tissues, and each tissue was sampled at three developmental stages based on Zadoks scale, which from the outer to inner were root at Z39, Z13, Z10, leaf at Z71, Z23, Z10, stem at Z65, Z32, Z30, spike at Z65, Z39, Z32, and grain at Z85, Z75, Z71. Numbers 1.00 to 8.00 represent the range of expression levels of *TaXTHs*, calculated as log2(RPKM + 1), from the lowest to the highest. I/II, III A and III B indicate the groups that TaXTHs belong to

The spatio-temporal expression of *TaXTHs* was analyzed in roots, stems, leaves, spikes and grains of wheat at different development stages using the wheat expression database. It was found that the genes in group III A had high expression levels in spikes and stems and low expression levels in grains, except for *TaXTH21-7A*, which had a high expression level in grains, indicating that *TaXTH21-7A* might be involved in grain development (Fig. 4). In group III B, the expression levels of *TaXTH26* genes were high only at the flowering stage of spikes, but had low or no expression in other tissues and stages. Other genes in this group had no obvious tissue expression specificity, and were expressed in all tissues. In addition, it was found that in group I/II, *TaXTH7-2A-1*, *TaXTH7-2A-2*, *TaXTH7-2B-1*, *TaXTH7-2D-1*, *TaXTH7-2D-2*, *TaXTH31-3A-1*, *TaXTH31-3B-1*, *TaXTH31-3B-3*, *TaXTH31-3D-1*, *TaXTH31-3D-2*, *TaXTH39-7A* and *TaXTH39-7B* were only highly expressed in roots, and had a low expression level or no expression in other tissues. These genes might be involved in root development.

Next, we analyzed the expression of *TaXTHs* under salt and drought stresses using RNA-Seq data. As shown in Figure S2, *TaXTH16-2A* and *TaXTH16-2D* were obviously upregulated, while *TaXTH16-2B*, *TaXTH17-1A*, *TaXTH17-1B*, *TaXTH17-1D*, *TaXTH11-7A*, *TaXTH11-7D*, *TaXTH15-7A*, *TaXTH15-7B*, *TaXTH15-7D*, *TaXTH30-6A*, *TaXTH31-3A-1*, *TaXTH31-3B-3*, *TaXTH31-3D-1*, *TaXTH31-3D-3* were downregulated by NaCl stress. In terms of PEG6000 treatment, *TaXTH16-2A*, *TaXTH16-2B*, and *TaXTH16-2D* were upregulated, while *TaXTH17-1A*, *TaXTH17-1B*, *TaXTH17-1D*, *TaXTH11-7D*, *TaXTH31-3B-2* and *TaXTH32-7D-2* were downregulated (Figure S2).

**Fig. 5:**
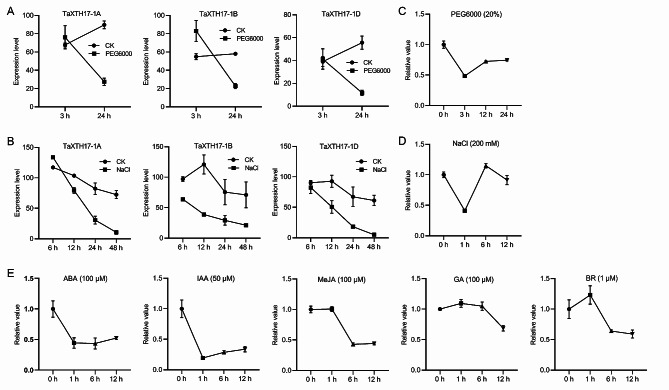
Expression patterns of *TaXTH17* in response to abiotic stresses and phytohormones. (**A**-**B**) Expression levels of *TaXTH17* genes in wheat under 15% PEG6000 (**A**) and 150 mM NaCl stresses (**B**). RNA-Seq data were from NCBI SRA database with accession numbers SRP145238 (**A**) and SRP062745 (**B**). (**C**-**E**) Expression levels of *TaXTH17-1D* determined by RT-qPCR under 20% PEG6000 (C), 200 mM NaCl (**D**), 100 µM ABA, 50 µM IAA, 100 µM MeJA, 100 µM GA and 1 µM BR (**E**), for the indicated time points. Total RNA was extracted from two weeks’ old wheat leaves subjected to the above mentioned abiotic stress and phytohormones. The wheat *GAPDH* gene was used as an internal control. All data represent means ± standard deviation

Since all three homeologs of *TaXTH17* were consistently induced by both NaCl and PEG6000 treatments (Figures S2 and 5A, 5B), we speculated that *TaXTH17-1A*, *1B* and *1D* share similar functions in response to salt and drought stresses, and randomly selected *TaXTH17-1D* for further study. Firstly, we determined the expression patterns of *TaXTH17-1D* in response to abiotic and hormone stresses using RT-qPCR. Under PEG6000, abscisic acid (ABA) and indole-3-acetic acid (IAA) treatments, the expression levels of *TaXTH17-1D* were rapidly downregulated and slightly recovered afterwards (Fig. 5C and E). During salt stress, the *TaXTH17-1D* transcripts decreased quickly at 1 h, recovered to similar levels as the control at 6 h, and then slightly decreased at 12 h (Fig. 5D). For methyl jasmonate (MeJA) and brassinosteroid (BR) treatment, the expression levels of *TaXTH17-1D* maintained to a certain degree at 1 h and dropped at 6 h (Fig. 5E). Under gibberellic acid (GA) treatment, the induction of *TaXTH17-1D* was relatively weak, and the mRNA levels slightly declined at 12 h (Fig. 5E).

### Subcellular localization of TaXTH17-1D protein

**Fig. 6:**
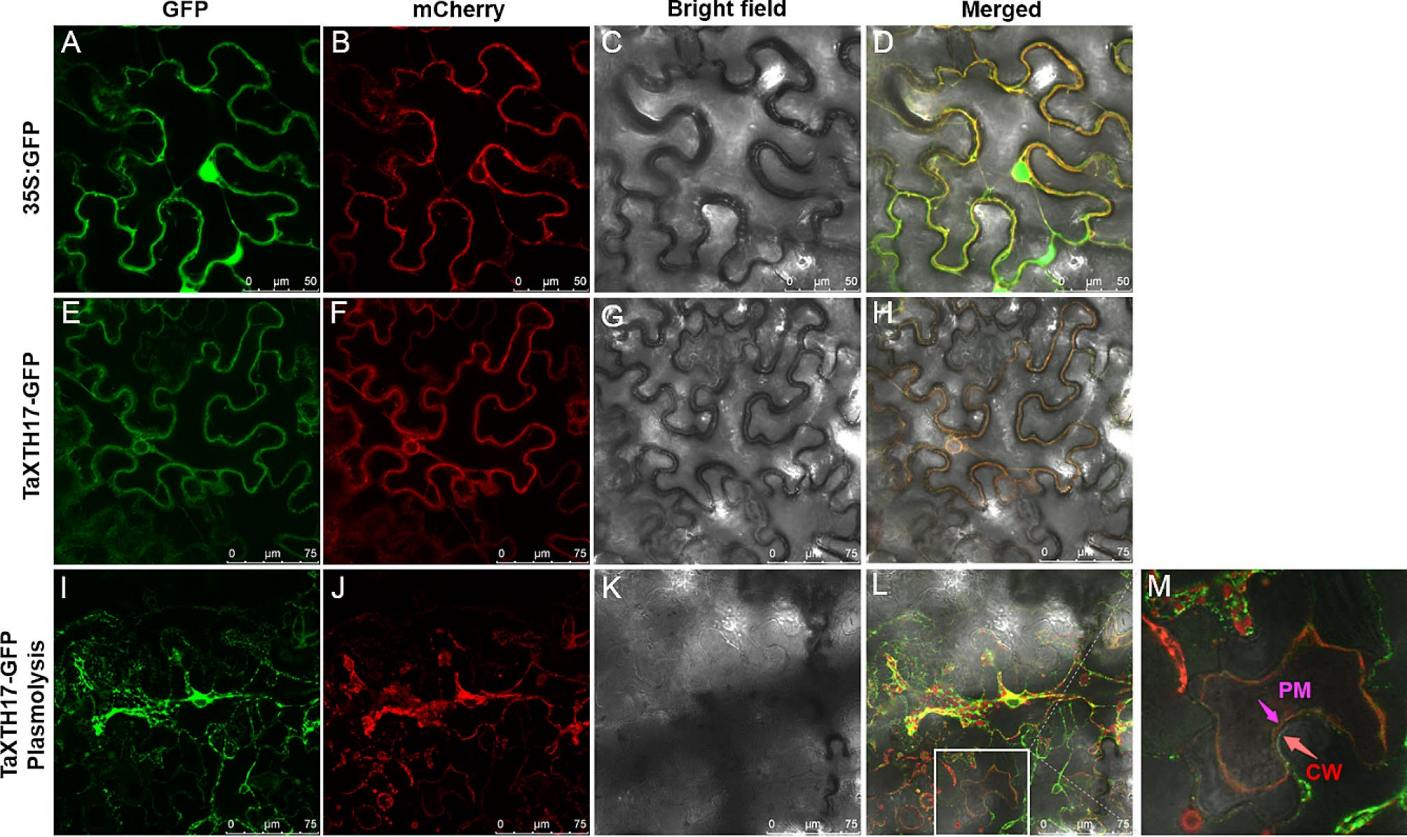
Subcellular localization of TaXTH17-1D. (**A**–**D**) Observation of *Nicotiana benthamiana* leaf cells co-expressing GFP protein and mcherry fused plasma membrane marker (pm-rk CD3-1007) under GFP channel (**A**), mcherry channel (**B**), bright-field (**C**), and with a merged view (**D**). (**E**–**H**) Observation of *Nicotiana benthamiana* leaf cells co-expressing GFP fused TaXTH17-1D and mcherry fused plasma membrane marker (pm-rk CD3-1007) under GFP channel (**E**), mcherry channel (**F**), bright-field (**G**), and with a merged view (**H**). (**I**–**L**) Observation of *Nicotiana benthamiana* leaf cells co-expressing GFP fused TaXTH17-1D and mcherry fused plasma membrane marker (pm-rk CD3-1007) under GFP channel (**I**), mcherry channel (**J**), bright-field (**K**), and with a merged view (**L**) after plasmolysis by treatment with 30% glycerol for 5 min. (**M**) An enlarged image of the selected region of (L) indicated by a white square frame. PM, plasma membrane; CW, cell wall

To gain insight into the distribution of TaXTH17-1D in the plant cell, TaXTH17-1D fused with GFP and the GFP control were transiently expressed in *N. benthamiana* leaves using *Agrobacterium* infiltration, and the fluorescence signal was observed using confocal microscopy. GFP signals from the transformation event of free GFP were visible in the nucleus, cytoplasm and around the cell periphery (Fig. 6A-D), while GFP signals from TaXTH17-GFP transformed plants were colocalized with the plasma membrane marker pm-rk CD3-1007 (Fig. 6E-H). Notably, GFP signals were also found around the nuclear membrane with a reticular structure, which was a sign of endoplasmic reticulum localization. These observations suggest that TaXTH17-1D is localized in the secretory pathway, including the endoplasmic reticulum and plasma membrane. To further determine whether TaXTH17-1D is localized to the cell wall, plasmolysis was performed and the fluorescence signals of TaXTH17-1D fused proteins were observed in the cell wall (Fig. 6I-M). These results indicate that TaXTH17-1D is localized to the secretory pathway and cell wall.

### *TaXTH17-1D* decreased yeast resistance to salt and drought

**Fig. 7:**
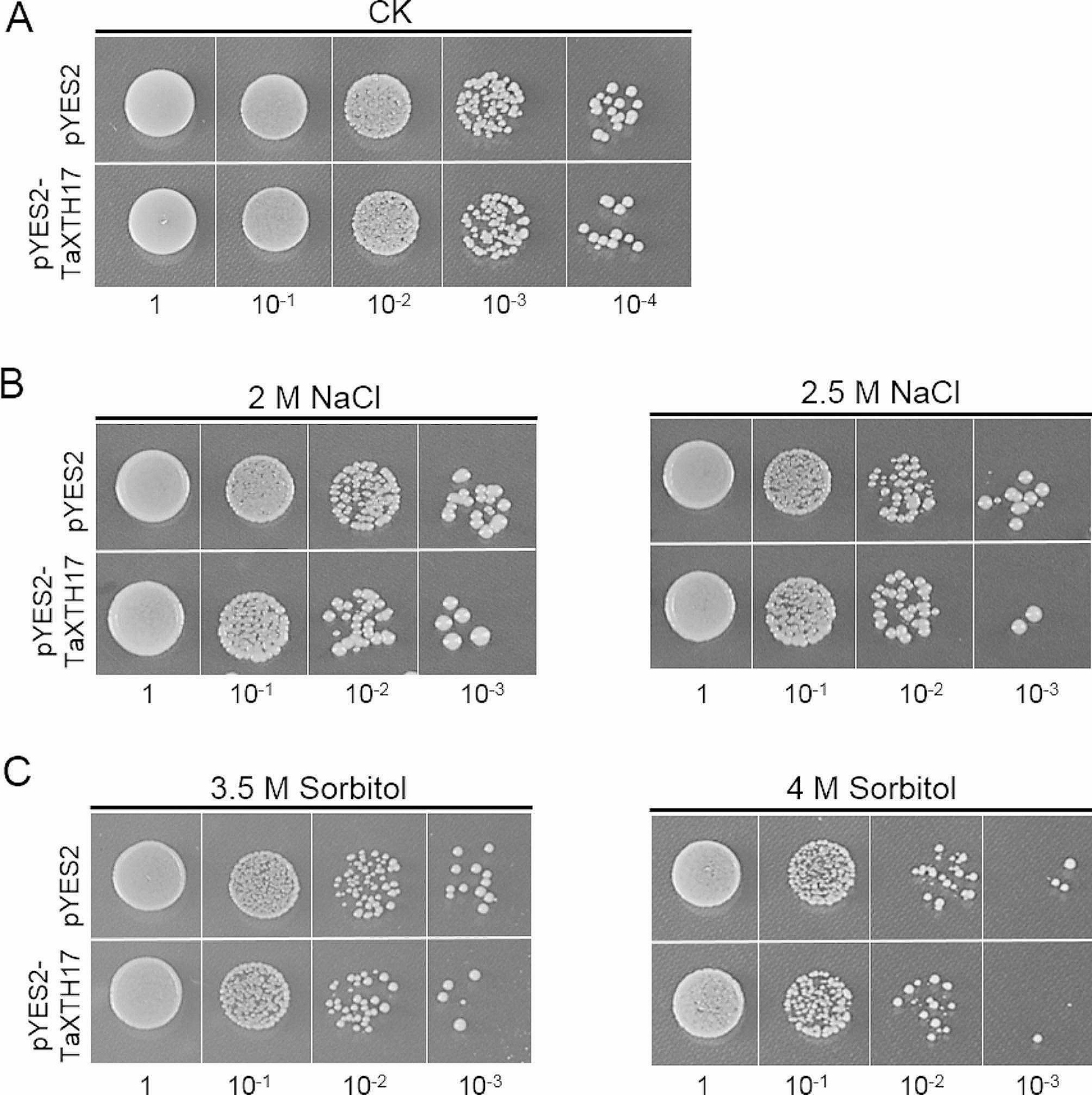
*TaXTH17-1D* confers salinity and drought tolerance in yeast. (**A**-**C**) Yeast cells transformed with pYES2-TaXTH17-1D and pYES2, respectively, grown on SD-Ura media after 24 hours’ growth in sterile water (CK) (**A**), 24 hours’ treatment with 2 M or 2.5 M NaCl (**B**), and 24 hours’ treatment with 3.5 M or 4 M Sorbitol (**C**). 1, 10 ^− 1^, 10 ^− 2^, 10 ^− 3^ and 10 ^− 4^ represent 10-fold serial dilutions of yeast cells

Next, we employed a yeast expression system to investigate the roles of *TaXTH17-1D* in response to salt and drought stresses. As shown in Fig. 7A, yeast cells transformed with pYES2-TaXTH17-1D recombinant vector exhibited a similar growth status to yeast cells with the pYES2 vector under normal growth conditions. However, when grown on media containing 2 M and 2.5 M NaCl, the number of yeast cells expressing *TaXTH17-1D* was noticeably lower than those harboring the empty vector (Fig. 7B). Similarly, fewer yeast cells transformed with pYES2-TaXTH17-1D survived under 3.5 M and 4 M sorbitol stresses compared with yeast carrying the empty pYES2 vector (Fig. 7C). These results suggest that *TaXTH17-1D* might play a negative role in plant resistance to salt and drought stresses.

### *TaXTH17-1D* negatively affects salt and drought tolerance in Arabidopsis

**Fig. 8:**
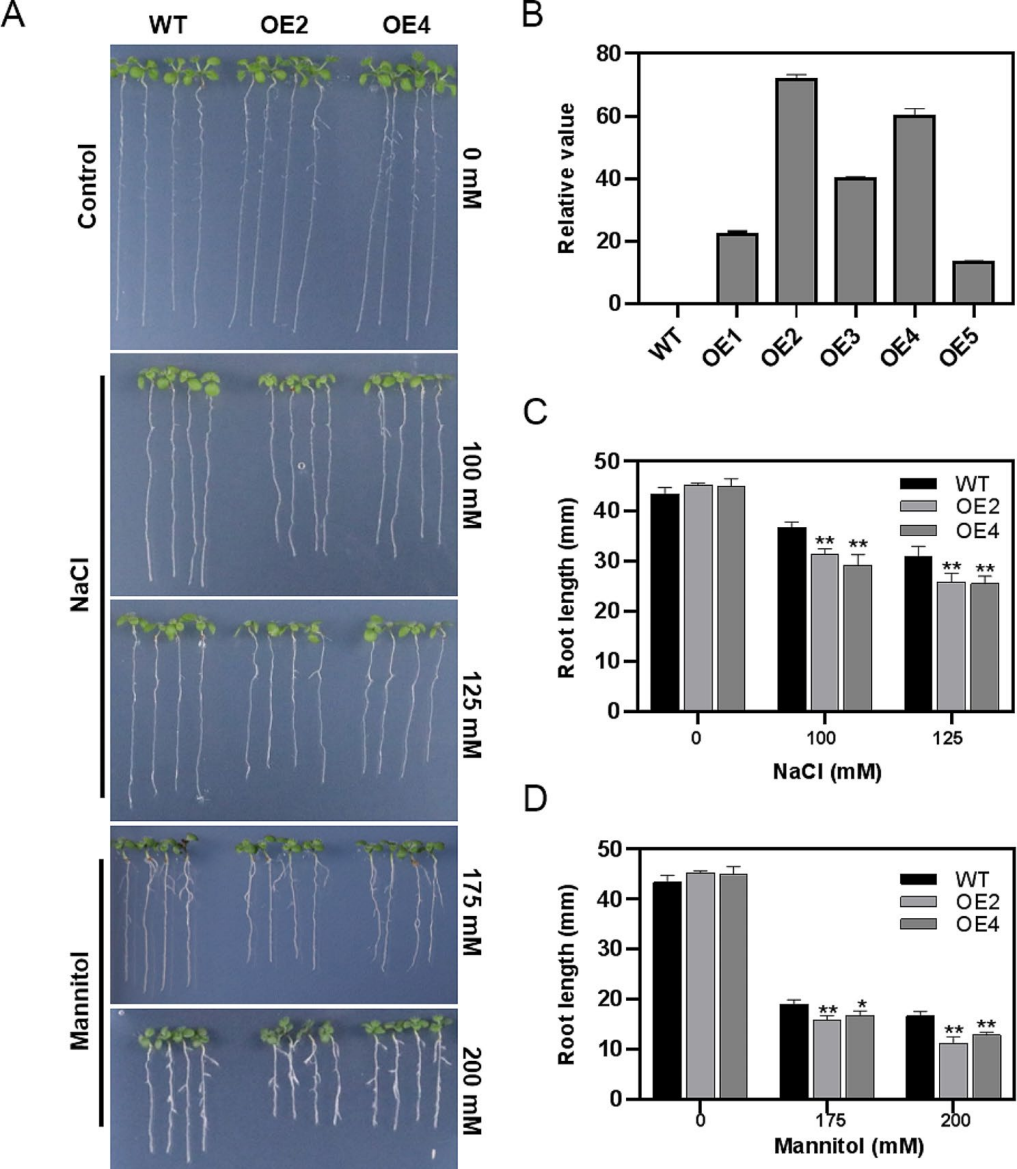
Overexpression of *TaXTH17-1D* reduced hypersensitivity to salt and drought stress in Arabidopsis seedlings. (**A**) Arabidopsis of the wild type (WT) and *TaXTH17-1D* overexpression seedlings (OE2 and OE4) grown on 1/2 MS, 1/2 MS supplemented with 100 mM and 125 mM NaCl as well as 175 mM and 200 mM mannitol. Photographs were taken after seven days’ growth of four days’ old seedlings on the media mentioned above. (**B**) The expression levels of *TaXTH17-1D* in WT and overexpression lines determined by RT-qPCR. (**C**) The primary root lengths of WT, OE2 and OE4 seedlings under 0, 100 and 125 mM NaCl conditions. (**D**) The primary root lengths of WT, OE2 and OE4 seedlings under 0, 175 and 200 mM mannitol conditions. Error bars indicate standard deviation. Asterisks indicate significant differences compared with the control by Student’s *t*-tests (* *P* < 0.05, ** *P* < 0.01)

To further explore the biological functions of *TaXTH17-1D* in plant resistance to salt and drought stresses, we constructed an overexpression vector with *TaXTH17-1D* under the control of the 35 S promoter and transformed it into Arabidopsis using the *Agrobacterium*-mediated method. Two independent T_3_ lines with high gene expression levels were selected for salt and drought tolerance assays (Fig. 8B). As shown in Fig. 8A, under normal growth conditions, no differences in primary root growth were observed between the wild type and *TaXTH17-1D* overexpression lines. However, under 100 mM and 125 mM NaCl, as well as 175 mM and 200 mM mannitol stress, the primary root lengths were significantly shorter for *TaXTH17-1D* overexpression lines compared with wild type plants (Fig. 8A, C and D). These results indicate that *TaXTH17-1D* negatively affects salt and drought stress tolerance in Arabidopsis.

### Silencing *TaXTH17* in wheat increased drought tolerance

**Fig. 9:**
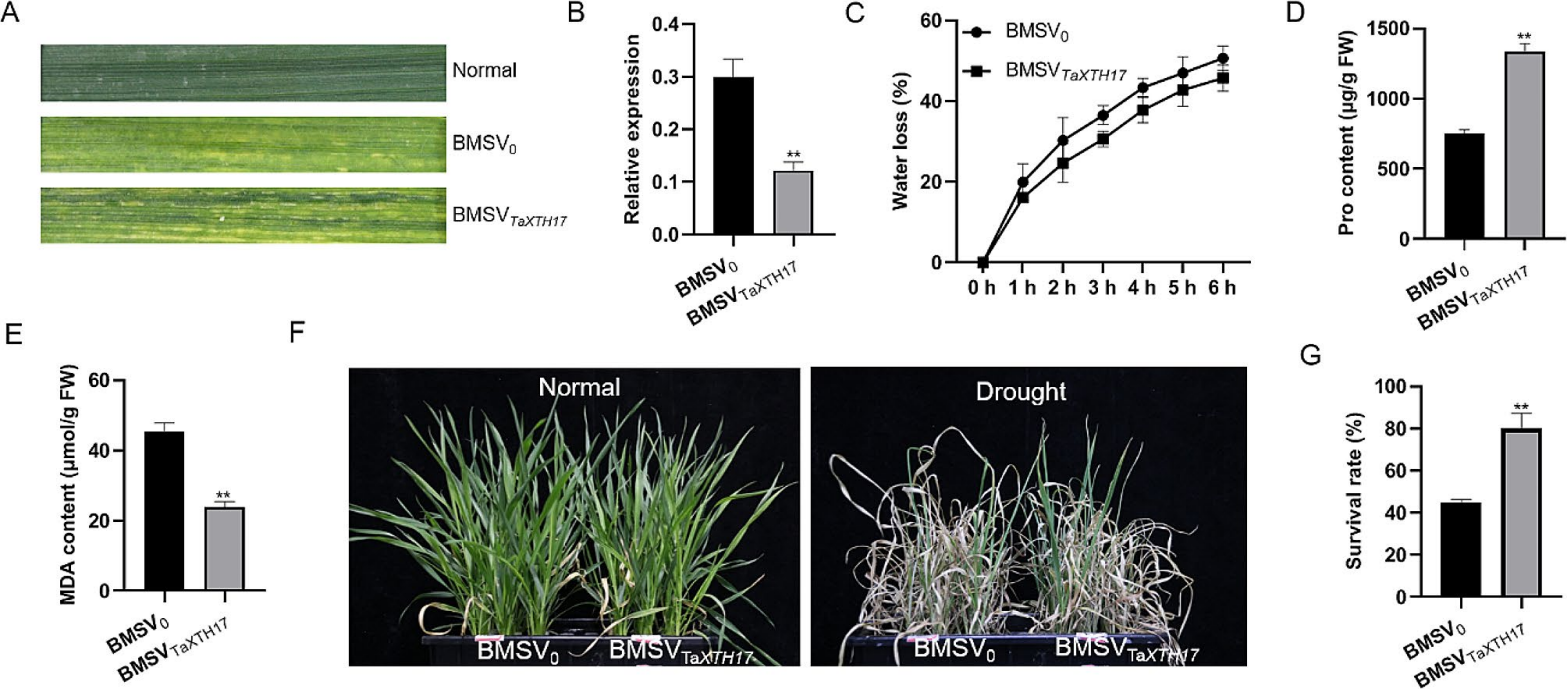
Silencing *TaXTH17* in wheat increased drought tolerance. (**A**) BSMV and BSMV-TaXTH17 inoculated wheat plants (BMSV_0_ and BMSV_*TaXTH17*_, respectively) showed virus infection phenotype on leaves about seven days after inoculation. (**B**) Gene expression levels of *TaXTH17* in BMSV_0_ and BMSV_*TaXTH17*_ wheat plants. (**C**) Water loss, (**D**) Proline content and (**E**) MDA content of BMSV_0_ and BMSV_*TaXTH17*_ wheat plants. (**F**) BMSV_0_ and BMSV_*TaXTH17*_ plants under normal and drought conditions. (**G**) Survival rate of BMSV_0_ and BMSV_*TaXTH17*_ wheat plants. Error bars indicate standard deviation. Asterisks indicate significant differences determined by Student’s *t*-tests (** *P* < 0.01)

Subsequently, we investigated whether *TaXTH17* functions in response to abiotic stress in wheat using barley stripe mosaic virus (BSMV)-mediated gene silencing. The BSMV-TaXTH17 construct and the BSMV (control) vector were inoculated into wheat leaves at the two-leaf stage, respectively. About two weeks after inoculation, when a widespread virus infection phenotype was observed on leaves (Fig. 9A), the transcript levels of *TaXTH17* in inoculated plants were determined by RT-qPCR. We found that the transcript abundance of *TaXTH17* in plants inoculated with BSMV-TaXTH17 (BSMV_TaXTH17_) was markedly decreased by approximately 59% compared with that in plants inoculated with BSMV vectors (BSMV_0_), indicating that the expression of *TaXTH17* was successfully suppressed in BSMV_TaXTH17_ wheat plants (Fig. 9B and S3). The successfully suppressed BSMV_TaXTH17_ plants and the BSMV_0_ plants were transferred to full-strength Hoagland solution with or without 20% PEG6000 for drought tolerance assays. Water loss tests revealed that the water loss of BSMV_TaXTH17_ wheat plants was less than that of BSMV_0_ plants (Fig. 9C). Proline content in BSMV_TaXTH17_ wheat plants increased by 76.4% compared to that in BSMV_0_ plants, while MDA content in BSMV_TaXTH17_ plants decreased by 47.6% compared to that in BSMV_0_ plants (Fig. 9D and E). Finally, BSMV_TaXTH17_ plants showed significantly higher survival rates than BSMV_0_ plants (Fig. 9F and G). These results indicate that *TaXTH17* plays a negative role in wheat response to drought.

## Discussion

The global identification of XTH genes will help to understand gene expression patterns and regulatory mechanisms that enable plants to withstand environmental stresses such as salt and drought. To date, numerous XTH genes have been identified in plants, including 33 genes in Arabidopsis [10], 29 in rice [13], 25 in tomato [14], 61 in soybean [15], 24 in barley [16] and 14 in tea [18]. In this study, 135 XTH genes were identified in hexaploid wheat (*Triticum aestivum* L., AABBDD), the number of which was considerably larger than that found in the species mentioned above. The main contributor to this substantial increase in wheat XTH genes is polyploidy, with a majority of wheat XTH genes having counterparts in the A, B and D genomes (Fig. 1, Table S1). Additionally, tandem duplication emerges as a secondary factor contributing to the expansion of the XTH gene family, accounting for 48.89% of the total XTH genes. Tandem duplication has also been implicated in the expansion of the XTH gene family in other plant species, such as Arabidopsis, soybean and barley [10, 15, 16].

Interaction network prediction revealed that the interaction proteins of TaXTHs include kinases, other enzymes and non-enzyme proteins (Fig. 3). To our knowledge, the report on XTH interacting proteins is very limited. Zhu et al. [23] performed protein interaction assays between AtXTH31 and 11 XTH genes expressed in Arabidopsis roots, and found that AtXTH31 interacted with AtXTH17; this interaction was further verified by coimmunoprecipitation assays. However, there is no empirical evidence regarding the interaction between XTHs and kinases. We performed a yeast two-hybrid screening for one wheat XTH and found that the screened interaction proteins also contained kinases (data not shown). Consequently, further investigation into the interaction between XTHs and kinases is warranted, potentially uncovering underlying mechanisms.

Gene expression patterns are intricately linked to gene functions. Hexaploid wheat genes predominantly exist as triplicate homeologs, and these triplicate homeologs undergo three potential evolutionary trajectories: retention of original or similar function, gene silencing, and functional diversification [29]. Gene expression pattern analyses demonstrated that the three homeologs of *TaXTH17* exhibit comparable spatio-temporal and stress-induced expression patterns (Figs. 4 and 5A and B). This similarity suggests that, during evolution, the functions of the three homeologs of *TaXTH17* have not undergone silencing or significant differentiation, instead maintaining similar functions. Consequently, to elucidate the function of *TaXTH17*, we randomly selected one of them, *TaXTH17-1D*, for further analysis. We found that *TaXTH17-1D* was suppressed by drought and salt stresses (Fig. 5C and D), indicating that TaXTH17 might play a negative role in drought and salt stresses in wheat. Extensive studies have revealed that various phytohormones could participate in the regulation of stress responses in plants [30]. In this study, we found that the expression of *TaXTH17-1D* was suppressed by several phytohormones including abscisic acid, auxin, methyl jasmonate and brassinosteroids (Fig. 5E). However, the trends of expression levels of *TaXTH17-1D* changed differently under the examined treatments of abiotic stresses and phytohormones. Under drought and salt stresses, the transcript levels of *TaXTH17-1D* decreased rapidly, which may be related to the remodeling of cell walls in order to adapt to environmental stresses and the inhibition of cell elongation under such stresses. Later, their expression levels recovered, possibly reflecting the necessity of *TaXTH17-1D* expression for the normal growth and development of plants [31]. ABA serves as the pivotal hormone that enables plants to adapt to environmental stress, while IAA is closely related to cell growth and development. The downregulation of *TaXTH17-1D* under ABA and IAA treatment may be related to the effects of these two hormones on cell wall extensibility [32]. The initial stable expression of *TaXTH17-1D* under MeJA and BR treatments may reflect the weak direct regulation of these two hormones on cell wall structure. The decrease in expression levels after 6 h may be related to plants response and adaptation mechanism to hormones. The weak induction effect of GA treatment on *TaXTH17-1D* may indicate that GA has no obvious direct regulation effect on *TaXTH17-1D*. Taken together, these different trends in expression levels suggest the complexity of the molecular mechanism involved and hence further investigation is warranted. Similarly, some *XTH* genes have been shown to be regulated by phytohormones such as abscisic acid, auxin, gibberellin and ethylene [31, 33–35]. Xu et al. [26] found that the brassinosteroid-responsive and salt-inducible *XTH19* and *XTH23* genes are involved in lateral root development under salt stress in Arabidopsis; both auxin and brasssinestroid signaling pathways regulate the physiological process of promoting lateral root development by regulating cell wall modification genes. Therefore, we speculate that *TaXTH17-1D* might play a role in salt and drought stresses through phytohormones including brassinosteroids and auxin.

Almost all TaXTH proteins contain signal peptides at the N-terminal (Table S1). Subcellular localization analyses in leaf epidermal cells of *Nicotiana benthamiana* revealed that TaXTH17-1D is located in the secretory pathway, including the endoplasmic reticulum and plasma membrane, as well as the cell wall (Fig. 6). Previous studies have shown that signal peptides guide XTH proteins to the cell membrane or cell wall. For instance, AtXTH30, AtXTH31 and AtXTH17 were found in the plasma membrane [23, 25], while ZmXTH1, DkXTH6 and DkXTH8 were localized to the cell wall [36–38]. Notably, the green fluorescence of ZmXTH1-GFP fusion protein was first detected in the secretory pathway (including the plasma membrane) in onion epidermal cells, and later it was observed in the cell wall after plasmolysis and pH change. In addition, a recent study illustrated that AtXTH11 and AtXTH33, both containing signal peptides, move towards the cell wall and plasma membrane via a conventional protein secretion (CPS) pathway, whereas XTH29, lacking a signal peptide, reaches the apoplast through an unconventional protein secretion (UPS) pathway. This suggests that probably all XTH proteins could reach the cell wall, but further detailed studies utilizing techniques such as plasmolysis, pH change and advanced fluorescence microscopy are required to verify this.

A substantial body of research has shown that *XTH* genes play important roles in plant growth, development and responses to various abiotic stresses. Their functions in abiotic stresses tolerance primarily encompass resistance to aluminum toxicity, salinity, drought and freezing. In this study, we specifically elucidated the role of *TaXTH17* in plant resistance to salt and drought stresses. Firstly, we found that fewer yeast cells transformed with *TaXTH17-1D* survived on media containing 2 M, 2.5 M NaCl and 3.5 M, 4 M sorbitol compared with control yeast cells (Fig. 7). Subsequently, to investigate the function of *TaXTH17-1D* in plants, we overexpressed *TaXTH17-1D* in Arabidopsis and found that Arabidopsis plants transformed with *TaXTH17-1D* exhibited hypersensitivity to salt and drought stresses, as evidenced by shorter primary root lengths on media containing 100 mM and 125 mM NaCl, as well as 175 mM and 200 mM mannitol compared with wild type plants (Fig. 8). Furthermore, our study demonstrated that wheat plants with silenced *TaXTH17* had enhanced drought resistance compared with control plants (Fig. 9). These findings collectively indicate that *TaXTH17* negatively affects plant responses to salt and drought stresses. Consequently, CRISPR-Cas9 gene editing technology or molecular breeding approaches could be harnessed to reduce the expression of *TaXTH17* gene or refine its expression pattern, thereby enhancing wheat resilience to drought and salt stresses. Wang et al. [39] uncovered that a nuclear-localized histone methyltransferase TaATX4 serves as a negative regulator of ABA signaling during drought stress. Notably, both ABA and water deficit can induce the expression of *TaATX4*. Furthermore, they successfully utilized CRISPR/Cas9 to create *TaATX4* mutant lines that exhibit heightened sensitivity to ABA and a remarkable enhancement in drought tolerance. This work suggests a practical strategy to improve wheat drought tolerance via CRISPR-Cas9 gene editing technology, and provides valuable germplasm resources for targeted breeding of drought-tolerant wheat cultivars. Previous studies found that *AtXTH30* plays a negative role in salt stress, mainly affecting the crystalline cellulose content and the depolymerization of microtubules [25]. In contrast, it was revealed that some *XTH* genes play positive roles in salt stress, such as *CaXTH3* and *PeXTH* [12, 20]. One possible explanation for their opposite roles in the same process is that they might possess divergent enzyme activities, which are xyloglucan endotransglucosylase (XET) activity and xyloglucan endohydrolase (XEH) activity. However, this hypothesis requires further experimental confirmation.

## Conclusions

In this study, we identified 135 wheat *XTH* genes, which were clustered into three evolutionary groups, namely groups I/II, III A and III B. These TaXTHs were unevenly distributed on wheat chromosomes, with segmental and tandem duplication being the main reasons for the expansion of the XTH family in wheat. Next, we predicted their interacting proteins and cis-acting elements related to abiotic stresses and phytohormone. Furthermore, we found that *TaXTH17*, which is potentially involved in stress responses, was induced by abiotic stresses and phytohormone treatments, and that TaXTH17 was localized in the secretory pathway and cell wall. Functional studies revealed that *TaXTH17* negatively affects plant tolerance to salt and drought stresses. Our results provide useful information that will help to clarify the functions and molecular mechanisms of *TaXTH* genes in the stress resistance of wheat.

## Materials and methods

### Identification and characteristics of *XTH* genes in wheat

Wheat XTH sequences were identified using the BioMart tool in the Ensembl Plants Database (https://plants.ensembl.org/index.html) [40] using two conserved domains of XTH as queries: PF00722 and PF06955. Next, XTH sequences with both of these two domains were found using a Venny tool (https://bioinfogp.cnb.csic.es/tools/venny/index.html) and designated as wheat XTHs. For protein sequences with the same gene identifier, only the sequence with the longest transcript was reserved. Meanwhile, sequences with mutations in the start codon and/or stop codon and incomplete sequencing were removed. Finally, 135 non-redundant TaXTH protein sequences were identified. The protein characteristics of the wheat *XTH* gene family were analyzed by ExPASy (https://web.expasy.org/protparam/), and the amino acid length and molecular weight of these genes were obtained. Subcellular localization was predicted by Plant-mPLoc (http://www.csbio.sjtu.edu.cn/bioinf/Cell-PLoc-2/); and signal peptides were predicted by SignalP 5.0 (http://www.cbs.dtu.dk/services/SignalP/).

### Phylogenetic relationship, gene structure and conserved motif analyses

The protein sequences of *XTH* gene family members from Arabidopsis and rice were obtained from the NCBI website (https://www.ncbi.nlm.nih.gov/). The Clustal W tool in MEGA7.0 was used to align the protein sequences of 33 *AtXTHs*, 29 *OsXTHs* and 135 *TaXTHs*. Subsequently, a phylogenetic tree was constructed based on the aligned sequences using the neighbor-joining method in MEGA 7.0, with the bootstrap parameter set to 1000. For gene stucture analysis, the whole-genome annotation information of wheat was downloaded from Ensemble Plants (http://plants.ensembl.org/info/data/ftp/index.html). Motif analysis was carried out using the MEME Suite tool (https://meme-suite.org/meme/tools/meme), with the number of motifs set to 10. The gene structure and conserved motifs were visualized through the Gene structure view (Advanced) feature in TBtools [41].

### Gene duplication analysis

Gene duplication analysis was performed using TBtools. Initially, the One Step MCScanX-Super Fast and related tools were used to generate a wheat gene linkedRegion file. Then, *TaXTH* gene pairs were extracted from the tandem gene pair file obtained above, producing a *TaXTH* gene pair file in TBtools. Finally, the wheat genome file was analyzed by the Fasta Stats tool to obtain the chromosome length information file. The collinearity visualization was accomplished by the Advanced Circos tool in Tbtools using the files generated above.

### Interaction network analysis

Orthologous pairs between TaXTHs and AtXTHs were identified through the OrthoVenn2 web tool (https://orthovenn2.bioinfotoolkits.net/home). Subsequently, the interaction networks of wheat *XTH* genes were investigated using the STRING (https://cn.string-db.org/) database based on the orthologous genes between wheat and Arabidopsis. The predicted interaction network was visualized through the Cytoscape software.

### Cis-element analysis

1500 bp sequences upstream of the start codon of genes were extracted from the wheat gene annotation file (Triticum_aestivum.IWGSC.50.gff3.gz) using the gff3 sequence extraction tool in Tbtools and regarded as gene promoter sequences. Then, the promoter sequences of *TaXTH* genes were obtained by searching all wheat gene promoter sequences using *TaXTH* gene IDs as queries via the Quick Fasta tool. The *TaXTH* promoter sequences were subjected to cis-element prediction using the PlantCare web tool (http://bioinformatics.psb.ugent.be/webtools/PlantCare/html/).

### Gene expression profile analyses

Spatio-temporal gene expression data of *TaXTH* genes were downloaded from the Wheat Expression Browser (http://www.wheat-expression.com/), including data on roots at Z10, Z13, Z39 stages, leaves at Z10, Z23, Z71 stages, stems at Z30, Z32, Z65 stages, spikes at Z32, Z39, Z65 stages, and grains at Z71, Z75, Z85 stages [42]. The heat map of *TaXTHs* gene expression was created using TBtools.

For gene expression analyses of *TaXTHs* under abiotic stresses, RNA-Seq data were downloaded from the NCBI SRA database with accession numbers SRP062745 (salt stress) and SRP145238 (drought stress). Transcript abundance was determined using Kallisto (version 0.46.0). For salt stress, one-week old wheat plants (Chinese Spring and Qing Mai 6) was subjected to 150 mM NaCl for 48 h [43]. The leaves were separately collected at 6 h, 12 h, 24 h and 48 h for transcriptome sequencing. For drought stress, Aikang 58 wheat plants at the two-leaf stage were treated with 15% PEG6000. The roots were individually collected at 3 h and 24 h for transcriptome sequencing.

### RT-qPCR

Total RNA was extracted using the RNAprep Pure Plant Kit (TIANGEN, Beijing, China) according to the manufacturer’s instructions. cDNA was synthesized using the NovoScript^®^ Plus All-in-one 1st Strand cDNA Synthesis SuperMix (gDNA Purge) (Novoprotein, Suzhou, China). ChamQ Universal SYBR qPCR Master mix (Vazyme, Nanjing, China) was used for RT-qPCR amplification in LightCycler 480 (Roche, Basel, Switzerland). The RT-qPCR conditions were as follows: 95 °C for 5 min, followed by 40 cycles of 95 °C for 15 s, 60 °C for 20 s, and 72 °C for 20 s. A melting curve analysis was performed at the end of the RT-qPCR reaction to verify the specificity of the primers used in this study. The wheat *GAPDH* gene was used as an internal reference gene for all RT-qPCR. The relative expression levels were calculated using the 2^−ΔCt^ method. The primers used are listed in Table S3.

### Subcellular localization analysis

The coding region of *TaXTH17-1D* without the stop codon was amplified using gene-specific primers and fused to the N-terminal of the green fluorescent protein (GFP) gene sequence under the control of the Cauliflower mosaic virus (CaMV) 35 S promoter. The TaXTH17-1D-GFP recombinant construct was then transformed into *Agrobaeterium tumefaeiens* strain GV3101, which was subsequently injected together with the plasma membrane marker (pm-rk CD3-1007) [44] into leaf epidermal cells of *Nicotiana benthamiana* via agro-infiltration. After two days of growth at 22 °C with a photoperiod of 16/8 h light/dark, the infiltrated leaves were visualized under a Leica TCS SP8 confocal laser-scanning microscope. Plasmolysis was performed with 30% glycerol for 5 min.

### Ectopic expression and stress tolerance assays in yeast

The coding region of *TaXTH17-1D* was amplified using gene-specific primers and inserted into the pYES2 vector using the *Eco*RI enzyme site. The resulting plasmid pYES2-TaXTH17-1D and the empty pYES2 vector were transformed into the yeast strain INVSc1 using the PEG/LiAc method. Positive transformants were screened on synthetic medium without Ura (SD/-Ura). The positive colonies were cultivated overnight at 30 °C in SD/-Ura medium, after which yeast cells were centrifuged and resuspended in IY/-Ura induction medium to achieve an OD600 of 0.4. After induction for 20 h until the OD600 reached 2.0, the yeast cells were subjected to NaCl (2 M and 2.5 M) and sorbitol (3.5 M and 4 M) stresses for 24 h, respectively, at 30 °C with shaking. The cells diluted with ddH_2_O were used as a control. After treatment, 10-fold serial dilutions of yeast cells were spotted onto SD/-Ura media and grown at 30 °C for 2–3 days.

### Genetic transformation and stress treatment in Arabidopsis

The coding region of *TaXTH17-1D* was amplified using gene-specific primers and constructed into the pCAMBIA1305-EGFP vector using the *Spe*I and *Bam*HI enzyme sites. The recombinant plasmid was then transformed into wild-type *Arabidopsis thaliana* (Col-0) using the floral dip method [45]. The transgenic plants were selected on 1/2 MS medium containing 40 mg L^− 1^ hygromycin and further confirmed by PCR. The expression levels of the transgene were assessed by RT-qPCR. T_3_ homozygous plants of transgenic Arabidopsis were used for subsequent analyses. For salt and drought stresses, five-days-old WT and transgenic overexpression lines were transferred onto 1/2 MS media supplemented with 0, 100, 125 mM NaCl and 175, 200 mM mannitol, respectively. Primary root lengths were measured, and photographs were taken seven days later.

### Virus-induced gene silencing

Virus-induced gene silencing (VIGS) of *TaXTH17* in wheat was conducted using barley stripe mosaic virus (BSMV) [46]. A 131 bp fragment of *TaXTH17* was amplified and constructed into the BSMV-γ plasmid to produce a BSMV-TaXTH17 vector, while BSMV-γ was used as a control. The protocol for transiently silencing *TaXTH17* expression in wheat leaves was previously described [47, 48]. After inoculation for 14 days, the phenotype of inoculated wheat seedlings was observed, and the leaves were collected for RNA extraction. Successfully silenced wheat seedlings carrying BSMV-TaXTH17 or BSMV-γ were transferred to full-strength Hoagland solution with 20% PEG6000 for 10 days and then re-watered for 7 days. For water loss assays, the fourth leaves of infected plants were detached and weighted immediately. They were then placed in a 25 °C growth chamber and weighted at designated time points. Water loss at each designated time points was calculated based on the initial weight of plants. Proline and MDA content were measured using a proline assay kit (PRO-1-Y; Suzhou Comin Biotech Co. Ltd) and a lipid peroxidation MDA assay kit (S0131S; Shanghai Beyotime Biotech Co. Ltd), respectively.

### Statistical analyses

Statistical analyses were performed using GraphPad Prism 7. Differences between WT and transgenic lines were evaluated using Student’s *t* tests.

### Electronic supplementary material

Below is the link to the electronic supplementary material.

Supplementary Material 1

## Data Availability

All data relevant to the results and analysis in this study are included in this article and its supplementary materials.
