# Supplementary material for: Genome-wide analysis of wheat xyloglucan endotransglucosylase/hydrolase (XTH) gene family revealed TaXTH17 involved in abiotic stress responses
Source: BMC Plant Biol. 2024 Jul 6;24:640. doi: 10.1186/s12870-024-05370-4 (PMC11227136; doi:10.1186/s12870-024-05370-4)
Supplement: Supplementary file 1 — Supplementary Material 1 [file 12870_2024_5370_MOESM1_ESM.doc]

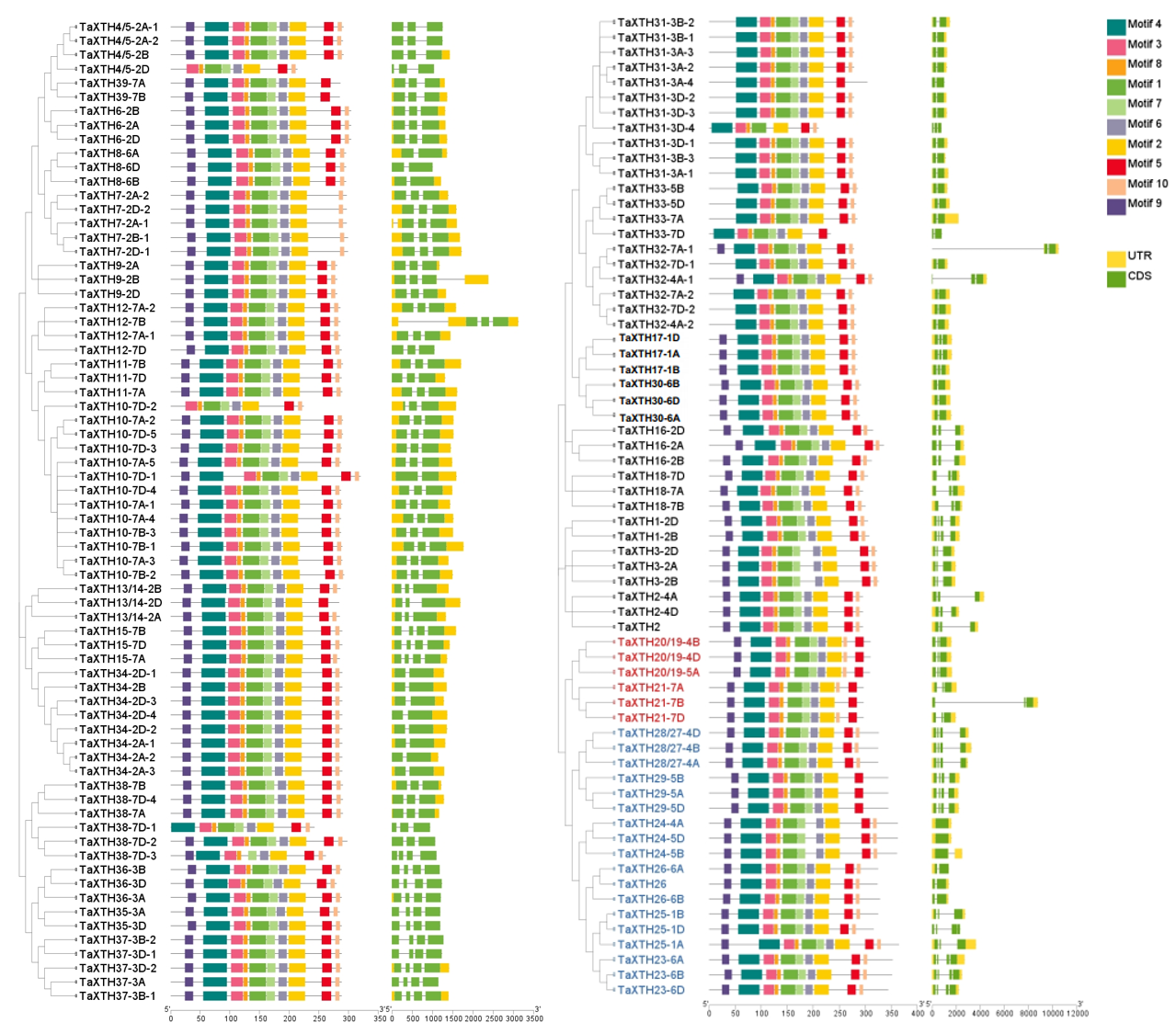


**Fig. S1** Conserved motifs and gene structures of TaXTHs. Genes in black, red and blue colors represent TaXTHs in groups I/II, III A and III B, respectively.


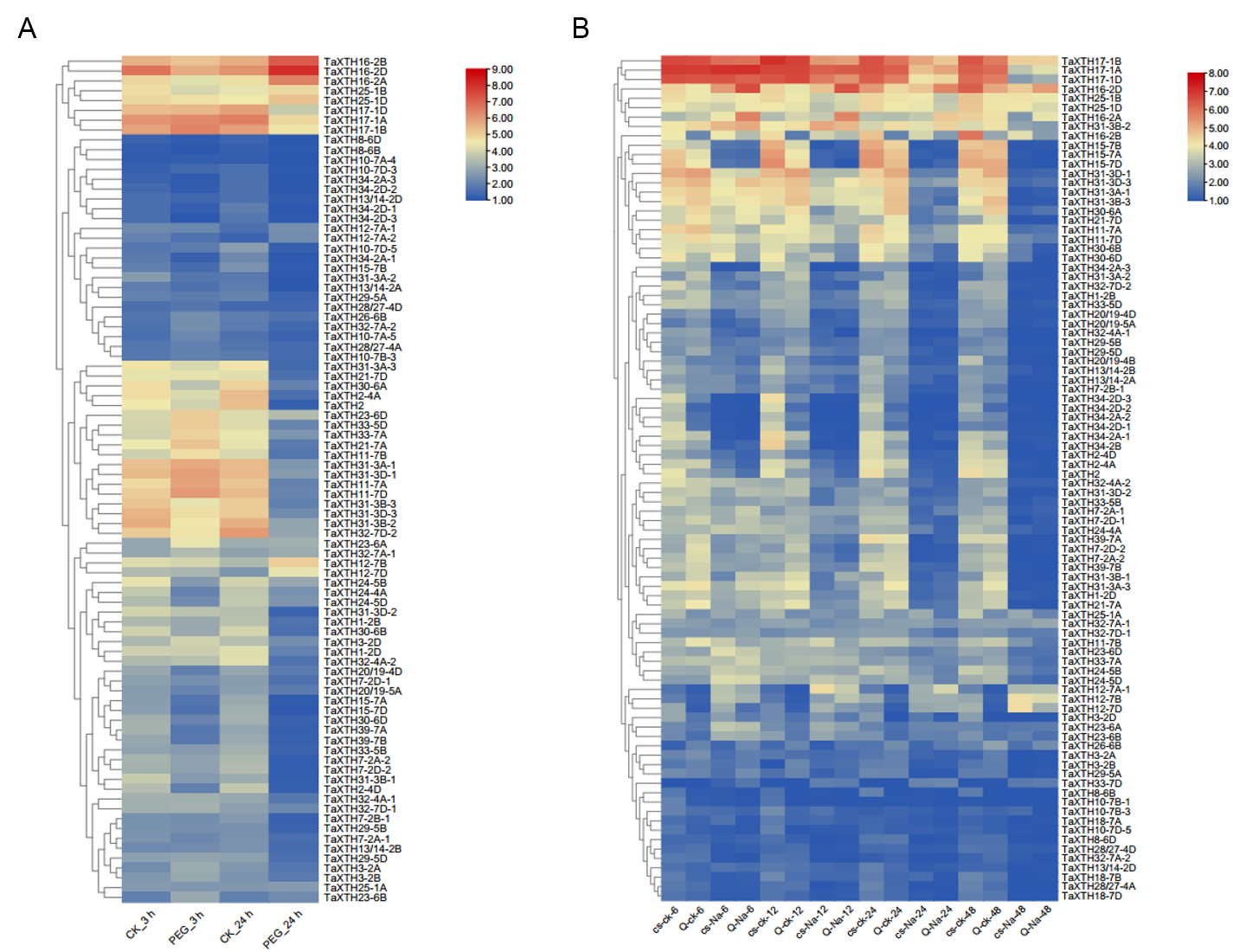


**Fig. S2** Heat maps showing the expression levels of *TaXTH* genes in wheat under drought (A) and NaCl (B) stresses. RNA-Seq data were downloaded from NCBI SRA database with accession numbers SRP145238 (A) and SRP062745 (B). Transcript abundance was determined using Kallisto. CK, the control group; PEG, 15% PEG6000 treatment; cs, Chinese spring; Q, QM-6; ck, the control group; Na, 150 mM NaCl treatment. 6, 12, 24, 48 represent that samples were collected at 6, 12, 24, 48 hours after salt stress. Numbers 1.00 to 9.00 and 1.00 to 8.00 represent the range of expression levels of TaXTHs, calculated as log2(RPKM+1), from the lowest to the highest.

**Fig. S3** Gene expression levels of *TaXTH17* in wild type and BMSV_0_ wheat plants. The results indicate that VIGS infection (empty vector, BSMV_0_) does not affect *TaXTH17* expression compared to the wild type. ns, no significant difference at *P* < 0.05 level determined by Student's *t*-test.

**Table S1.**  Information on wheat XTH genes identified in wheat

| Gene name | Gene ID | Peptide length (aa) | Molecular weight (kDa) | SignalP | Subcellular localization |
| --- | --- | --- | --- | --- | --- |
| *TaXTH1-2B* | TraesCS2B02G244500.1 | 306 | 34.23543 | 22 | Cell wall |
| *TaXTH1-2D* | TraesCS2D02G224300.1 | 304 | 33.87201 | 19 | Cell wall |
| *TaXTH2* | TraesCSU02G175000.1 | 294 | 33.51952 | 22 | Cell wall Cytoplasm |
| *TaXTH2-4A* | TraesCS4A02G172600.1 | 294 | 33.51956 | 22 | Cell wall Cytoplasm |
| *TaXTH2-4D* | TraesCS4D02G142800.1 | 294 | 33.47555 | 22 | Cell wall Cytoplasm |
| *TaXTH3-2A* | TraesCS2A02G484100.2 | 322 | 35.36432 | 28 | Cell wall |
| *TaXTH3-2B* | TraesCS2B02G510200.1 | 325 | 35.79577 | 28 | Cell wall |
| *TaXTH3-2D* | TraesCS2D02G484000.1 | 321 | 35.33528 | 28 | Cell wall |
| *TaXTH4/5-2A-1* | TraesCS2A02G047100.1 | 291 | 33.55771 | 29 | Cell wall |
| *TaXTH4/5-2A-2* | TraesCS2A02G047200.1 | 290 | 33.32636 | 28 | Cell wall |
| *TaXTH4/5-2B* | TraesCS2B02G059000.1 | 291 | 33.47678 | 29 | Cell wall |
| *TaXTH4/5-2D* | TraesCS2D02G045100.1 | 213 | 24.76275 | — | Cell wall |
| *TaXTH6-2A* | TraesCS2A02G434300.1 | 303 | 33.59086 | 23 | Cell wall |
| *TaXTH6-2B* | TraesCS2B02G455400.1 | 303 | 33.68583 | 23 | Cell wall |
| *TaXTH6-2D* | TraesCS2D02G432100.1 | 303 | 33.66803 | 28 | Cell wall |
| *TaXTH7-2A-1* | TraesCS2A02G433600.1 | 296 | 33.72483 | 29 | Cell wall |
| *TaXTH7-2A-2* | TraesCS2A02G433700.1 | 296 | 33.89085 | 29 | Cell wall |
| *TaXTH7-2B-1* | TraesCS2B02G454600.1 | 298 | 33.78893 | 31 | Cell wall |
| *TaXTH7-2D-1* | TraesCS2D02G431600.1 | 298 | 33.72291 | 31 | Cell wall |
| *TaXTH7-2D-2* | TraesCS2D02G431700.1 | 296 | 33.92998 | 29 | Cell wall |
| *TaXTH8-6A* | TraesCS6A02G337600.1 | 295 | 33.85105 | 31 | Cell wall |
| *TaXTH8-6B* | TraesCS6B02G368300.1 | 295 | 34.00513 | 31 | Cell wall |
| *TaXTH8-6D* | TraesCS6D02G318500.1 | 295 | 33.88704 | 31 | Cell wall |
| *TaXTH9-2A* | TraesCS2A02G433800.1 | 280 | 31.10565 | 26 | Cell wall |
| *TaXTH9-2B* | TraesCS2B02G454800.1 | 280 | 30.99759 | 26 | Cell wall |
| *TaXTH9-2D* | TraesCS2D02G431800.1 | 280 | 31.18179 | 26 | Cell wall |
| *TaXTH10-7A-1* | TraesCS7A02G426100.1 | 289 | 32.12225 | 21 | Cell wall Cytoplasm |
| *TaXTH10-7A-2* | TraesCS7A02G426600.1 | 290 | 32.24339 | 20 | Cell wall Cytoplasm |
| *TaXTH10-7A-3* | TraesCS7A02G426700.1 | 289 | 31.96891 | 18 | Cell wall Cytoplasm |
| *TaXTH10-7A-4* | TraesCS7A02G426900.1 | 286 | 31.85481 | 18 | Cell wall Cytoplasm |
| *TaXTH10-7A-5* | TraesCS7A02G427000.1 | 286 | 31.99409 | 18 | Cell wall Cytoplasm |
| *TaXTH10-7B-1* | TraesCS7B02G326900.1 | 289 | 32.19834 | 21 | Cell wall Cytoplasm |
| *TaXTH10-7B-2* | TraesCS7B02G327000.1 | 292 | 32.40555 | 21 | Cell wall |
| *TaXTH10-7B-3* | TraesCS7B02G327200.1 | 286 | 31.84486 | 18 | Cell wall Cytoplasm |
| *TaXTH10-7D-1* | TraesCS7D02G418600.1 | 319 | 35.9489 | 21 | Cell wall |
| *TaXTH10-7D-2* | TraesCS7D02G418700.1 | 223 | 25.04295 | — | Cell wall |
| *TaXTH10-7D-3* | TraesCS7D02G419100.1 | 288 | 32.17917 | 20 | Cell wall Cytoplasm |
| *TaXTH10-7D-4* | TraesCS7D02G419200.1 | 286 | 31.85494 | 18 | Cell wall Cytoplasm |
| *TaXTH10-7D-5* | TraesCS7D02G419300.1 | 290 | 32.31447 | 20 | Cell wall Cytoplasm |
| *TaXTH11-7A* | TraesCS7A02G427600.1 | 289 | 31.88897 | 20 | Cell wall |
| *TaXTH11-7B* | TraesCS7B02G327700.1 | 290 | 32.03619 | 20 | Cell wall |
| *TaXTH11-7D* | TraesCS7D02G419900.1 | 287 | 31.7689 | 20 | Cell wall |
| *TaXTH12-7A-1* | TraesCS7A02G427100.1 | 285 | 31.55117 | 26 | Cell wall |
| *TaXTH12-7A-2* | TraesCS7A02G427300.1 | 285 | 31.49408 | 26 | Cell wall |
| *TaXTH12-7B* | TraesCS7B02G327400.1 | 285 | 31.56224 | 26 | Cell wall |
| *TaXTH12-7D* | TraesCS7D02G419400.1 | 287 | 31.70655 | 28 | Cell wall |
| *TaXTH13/14-2A* | TraesCS2A02G089000.1 | 284 | 31.22978 | 23 | Cell wall |
| *TaXTH13/14-2B* | TraesCS2B02G104700.1 | 285 | 31.47715 | 24 | Cell wall |
| *TaXTH13/14-2D* | TraesCS2D02G086800.1 | 283 | 31.36688 | 22 | Cell wall |
| *TaXTH15-7A* | TraesCS7A02G331200.1 | 285 | 31.61179 | 25 | Cell wall |
| *TaXTH15-7B* | TraesCS7B02G242900.1 | 289 | 32.07225 | 25 | Cell wall |
| *TaXTH15-7D* | TraesCS7D02G339400.1 | 289 | 32.06816 | 25 | Cell wall |
| *TaXTH16-2A* | TraesCS2A02G433500.1 | 312 | 36.3276 | 22 | Cell wall |
| *TaXTH16-2B* | TraesCS2B02G454500.1 | 311 | 33.61869 | 20 | Cell wall |
| *TaXTH16-2D* | TraesCS2D02G431500.1 | 314 | 34.05309 | 27 | Cell wall |
| *TaXTH17-1A* | TraesCS1A02G396200.1 | 283 | 31.01028 | 24 | Cell wall |
| *TaXTH17-1B* | TraesCS1B02G424500.1 | 283 | 30.97334 | 24 | Cell wall |
| *TaXTH17-1D* | TraesCS1D02G404300.1 | 283 | 31.07134 | 24 | Cell wall |
| *TaXTH18-7A* | TraesCS7A02G427200.1 | 295 | 33.65593 | 22 | Cell wall |
| *TaXTH18-7B* | TraesCS7B02G327500.1 | 299 | 34.0444 | 23 | Cell wall |
| *TaXTH18-7D* | TraesCS7D02G419600.1 | 304 | 34.26761 | 27 | Cell wall |
| *TaXTH20/19-4B* | TraesCS4B02G383000.1 | 309 | 34.62507 | 37 | Cell wall |
| *TaXTH20/19-4D* | TraesCS4D02G358700.1 | 309 | 34.45884 | 37 | Cell wall |
| *TaXTH20/19-5A* | TraesCS5A02G548500.1 | 308 | 34.5739 | 36 | Cell wall |
| *TaXTH21-7A* | TraesCS7A02G363000.1 | 296 | 33.43857 | 24 | Cell wall |
| *TaXTH21-7B* | TraesCS7B02G265000.1 | 296 | 33.85616 | 24 | Cell wall |
| *TaXTH21-7D* | TraesCS7D02G360100.1 | 296 | 33.694 | 24 | Cell wall |
| *TaXTH23-6A* | TraesCS6A02G266100.1 | 351 | 38.06291 | 23 | Cell wall |
| *TaXTH23-6B* | TraesCS6B02G293500.1 | 350 | 38.4204 | 28 | Cell wall |
| *TaXTH23-6D* | TraesCS6D02G248500.1 | 343 | 37.59536 | 23 | Cell wall |
| *TaXTH24-4A* | TraesCS4A02G321800.1 | 361 | 39.92381 | 24 | Cell wall |
| *TaXTH24-5B* | TraesCS5B02G556500.1 | 360 | 39.81768 | 24 | Cell wall |
| *TaXTH24-5D* | TraesCS5D02G552800.1 | 361 | 39.9107 | 24 | Cell wall |
| *TaXTH25-1A* | TraesCS1A02G195900.1 | 363 | 39.56139 | 22 | Cell wall |
| *TaXTH25-1B* | TraesCS1B02G210500.1 | 323 | 35.40179 | 22 | Cell wall |
| *TaXTH25-1D* | TraesCS1D02G199300.1 | 315 | 34.57592 | 22 | Cell wall |
| *TaXTH26* | TraesCSU02G018400.1 | 322 | 35.94961 | 22 | Cell wall |
| *TaXTH26-6A* | TraesCS6A02G088000.1 | 324 | 36.15783 | 22 | Cell wall |
| *TaXTH26-6B* | TraesCS6B02G110400.1 | 327 | 36.49422 | 20 | Cell wall |
| *TaXTH28/27-4A* | TraesCS4A02G073300.2 | 299 | 32.71765 | 21 | Cell wall |
| *TaXTH28/27-4B* | TraesCS4B02G229500.1 | 323 | 35.719 | 21 | Cell wall |
| *TaXTH28/27-4D* | TraesCS4D02G230600.1 | 325 | 35.83926 | 21 | Cell wall |
| *TaXTH29-5A* | TraesCS5A02G203300.1 | 343 | 37.7655 | 34 | Cell wall |
| *TaXTH29-5B* | TraesCS5B02G202000.1 | 343 | 37.7115 | 34 | Cell wall |
| *TaXTH29-5D* | TraesCS5D02G209700.1 | 343 | 37.73348 | 34 | Cell wall |
| *TaXTH30-6A* | TraesCS6A02G408300.1 | 288 | 32.35718 | 25 | Cell wall |
| *TaXTH30-6B* | TraesCS6B02G454200.1 | 291 | 32.55128 | 28 | Cell wall |
| *TaXTH30-6D* | TraesCS6D02G392100.1 | 287 | 32.14887 | 21 | Cell wall |
| *TaXTH31-3A-1* | TraesCS3A02G093400.1 | 278 | 31.10566 | 21 | Cell wall |
| *TaXTH31-3A-2* | TraesCS3A02G093500.1 | 277 | 31.46629 | 21 | Cell wall |
| *TaXTH31-3A-3* | TraesCS3A02G093600.1 | 278 | 31.50837 | 21 | Cell wall |
| *TaXTH31-3A-4* | TraesCS3A02G093700.1 | 303 | 34.22858 | 21 | Cell wall |
| *TaXTH31-3B-1* | TraesCS3B02G108600.1 | 277 | 31.32433 | 21 | Cell wall |
| *TaXTH31-3B-2* | TraesCS3B02G108700.1 | 277 | 31.32323 | 21 | Cell wall |
| *TaXTH31-3B-3* | TraesCS3B02G108800.1 | 278 | 31.18868 | 21 | Cell wall |
| *TaXTH31-3D-1* | TraesCS3D02G093700.1 | 278 | 31.20771 | 21 | Cell wall |
| *TaXTH31-3D-2* | TraesCS3D02G093800.1 | 277 | 31.18698 | 21 | Cell wall |
| *TaXTH31-3D-3* | TraesCS3D02G093900.1 | 277 | 31.25918 | 21 | Cell wall |
| *TaXTH31-3D-4* | TraesCS3D02G094000.1 | 209 | 23.72137 | — | Cell wall |
| *TaXTH32-4A-1* | TraesCS4A02G402200.1 | 282 | 34.77834 | 24 | Cell wall |
| *TaXTH32-4A-2* | TraesCS4A02G402300.1 | 281 | 31.17579 | 21 | Cell wall |
| *TaXTH32-7A-1* | TraesCS7A02G103500.1 | 278 | 30.63751 | 20 | Cell wall |
| *TaXTH32-7A-2* | TraesCS7A02G103600.1 | 277 | 30.74636 | — | Cell wall |
| *TaXTH32-7D-1* | TraesCS7D02G097300.1 | 281 | 30.95181 | 23 | Cell wall |
| *TaXTH32-7D-2* | TraesCS7D02G097400.1 | 281 | 31.10769 | 21 | Cell wall |
| *TaXTH33-5B* | TraesCS5B02G028800.1 | 285 | 31.47921 | 23 | Cell wall |
| *TaXTH33-5D* | TraesCS5D02G038400.1 | 281 | 31.11867 | 20 | Cell wall |
| *TaXTH33-7A* | TraesCS7A02G063300.1 | 283 | 31.31894 | 21 | Cell wall |
| *TaXTH33-7D* | TraesCS7D02G058200.1 | 233 | 26.17332 | — | Cell wall |
| *TaXTH34-2A-1* | TraesCS2A02G498200.1 | 289 | 32.06409 | 23 | Cell wall |
| *TaXTH34-2A-2* | TraesCS2A02G498300.1 | 289 | 32.12016 | 23 | Cell wall |
| *TaXTH34-2A-3* | TraesCS2A02G498400.1 | 289 | 32.12016 | 23 | Cell wall |
| *TaXTH34-2B* | TraesCS2B02G526500.1 | 289 | 32.08806 | 23 | Cell wall |
| *TaXTH34-2D-1* | TraesCS2D02G498400.1 | 289 | 32.05812 | 23 | Cell wall |
| *TaXTH34-2D-2* | TraesCS2D02G498500.2 | 289 | 32.03401 | 23 | Cell wall |
| *TaXTH34-2D-3* | TraesCS2D02G498600.1 | 289 | 32.19218 | 23 | Cell wall |
| *TaXTH34-2D-4* | TraesCS2D02G498700.1 | 289 | 32.19218 | 23 | Cell wall |
| *TaXTH35-3A* | TraesCS3A02G083900.1 | 284 | 31.29644 | 24 | Cell wall |
| *TaXTH35-3D* | TraesCS3D02G084000.1 | 288 | 31.86415 | 28 | Cell wall |
| *TaXTH36-3A* | TraesCS3A02G084000.1 | 287 | 31.71165 | 31 | Cell wall |
| *TaXTH36-3B* | TraesCS3B02G099100.1 | 288 | 31.84688 | 31 | Cell wall |
| *TaXTH36-3D* | TraesCS3D02G084100.1 | 279 | 30.79464 | 27 | Cell wall |
| *TaXTH37-3A* | TraesCS3A02G308800.1 | 287 | 32.45795 | 23 | Cell wall |
| *TaXTH37-3B-1* | TraesCS3B02G151700.1 | 287 | 32.27182 | 23 | Cell wall |
| *TaXTH37-3B-2* | TraesCS3B02G152000.1 | 287 | 32.45793 | 23 | Cell wall |
| *TaXTH37-3D-1* | TraesCS3D02G133300.1 | 287 | 32.42687 | 23 | Cell wall |
| *TaXTH37-3D-2* | TraesCS3D02G133800.2 | 287 | 32.5131 | 26 | Cell wall |
| *TaXTH38-7A* | TraesCS7A02G194300.1 | 290 | 32.8656 | 23 | Cell wall |
| *TaXTH38-7B* | TraesCS7B02G099900.1 | 290 | 32.69435 | 23 | Cell wall |
| *TaXTH38-7D-1* | TraesCS7D02G195300.1 | 242 | 27.84531 | — | Cell wall |
| *TaXTH38-7D-2* | TraesCS7D02G195600.1 | 297 | 33.98648 | 25 | Cell wall |
| *TaXTH38-7D-3* | TraesCS7D02G195800.1 | 261 | 29.74262 | 25 | Cell wall |
| *TaXTH38-7D-4* | TraesCS7D02G195900.1 | 290 | 32.85256 | 23 | Cell wall |
| *TaXTH39-7A* | TraesCS7A02G463800.1 | 285 | 32.02103 | 27 | Cell wall |
| *TaXTH39-7B* | TraesCS7B02G364300.1 | 284 | 32.01804 | 27 | Cell wall |

**Table S2.** Cis-elements in the promoter regions of wheat XTH genes

| Gene name | TGA-element | TC-rich repeats | TATC-box | LTR | TCA-element | ABRE | ARE | TGACG-motif | GC-motif | MBS | WUN-motif | Total number of elements |
| --- | --- | --- | --- | --- | --- | --- | --- | --- | --- | --- | --- | --- |
| *TaXTH1-2B* | 0 | 0 | 1 | 2 | 1 | 1 | 2 | 8 | 0 | 3 | 0 | 18 |
| *TaXTH1-2D* | 0 | 0 | 1 | 0 | 0 | 1 | 0 | 6 | 1 | 3 | 0 | 12 |
| *TaXTH2* | 1 | 0 | 0 | 1 | 0 | 1 | 1 | 2 | 0 | 1 | 0 | 7 |
| *TaXTH2-4A* | 0 | 0 | 0 | 0 | 0 | 1 | 0 | 4 | 0 | 1 | 0 | 6 |
| *TaXTH2-4D* | 0 | 0 | 1 | 2 | 0 | 2 | 2 | 4 | 2 | 0 | 0 | 13 |
| *TaXTH3-2A* | 0 | 0 | 0 | 1 | 0 | 5 | 2 | 2 | 1 | 2 | 0 | 13 |
| *TaXTH3-2B* | 2 | 0 | 0 | 0 | 0 | 7 | 0 | 6 | 1 | 1 | 0 | 17 |
| *TaXTH3-2D* | 2 | 0 | 1 | 1 | 6 | 0 | 4 | 1 | 2 | 0 | 0 | 17 |
| *TaXTH4/5-2A-1* | 1 | 0 | 1 | 0 | 0 | 0 | 1 | 2 | 0 | 2 | 0 | 7 |
| *TaXTH4/5-2A-2* | 0 | 0 | 0 | 0 | 0 | 1 | 1 | 8 | 0 | 2 | 0 | 12 |
| *TaXTH4/5-2B* | 1 | 0 | 1 | 0 | 0 | 1 | 2 | 2 | 0 | 2 | 0 | 9 |
| *TaXTH4/5-2D* | 0 | 0 | 0 | 1 | 0 | 1 | 1 | 6 | 1 | 2 | 0 | 12 |
| *TaXTH6-2A* | 0 | 0 | 0 | 0 | 0 | 2 | 1 | 2 | 0 | 2 | 0 | 7 |
| *TaXTH6-2B* | 1 | 0 | 0 | 0 | 1 | 3 | 1 | 2 | 1 | 2 | 0 | 11 |
| *TaXTH6-2D* | 2 | 0 | 0 | 1 | 0 | 2 | 3 | 0 | 2 | 1 | 0 | 11 |
| *TaXTH7-2A-1* | 0 | 0 | 0 | 0 | 0 | 2 | 2 | 0 | 0 | 1 | 0 | 5 |
| *TaXTH7-2A-2* | 0 | 0 | 1 | 0 | 0 | 3 | 3 | 6 | 0 | 2 | 0 | 15 |
| *TaXTH7-2B-1* | 0 | 0 | 0 | 0 | 0 | 4 | 2 | 2 | 0 | 0 | 0 | 8 |
| *TaXTH7-2D-1* | 1 | 0 | 2 | 2 | 0 | 5 | 1 | 12 | 1 | 3 | 0 | 27 |
| *TaXTH7-2D-2* | 0 | 0 | 0 | 1 | 0 | 1 | 1 | 4 | 0 | 1 | 0 | 8 |
| *TaXTH8-6A* | 0 | 0 | 0 | 0 | 0 | 1 | 1 | 0 | 1 | 1 | 0 | 4 |
| *TaXTH8-6B* | 0 | 0 | 0 | 0 | 0 | 1 | 0 | 0 | 0 | 0 | 0 | 1 |
| *TaXTH8-6D* | 1 | 1 | 0 | 0 | 0 | 8 | 1 | 4 | 0 | 0 | 0 | 15 |
| *TaXTH9-2A* | 1 | 0 | 3 | 0 | 0 | 3 | 2 | 0 | 0 | 0 | 0 | 9 |
| *TaXTH9-2B* | 1 | 0 | 0 | 1 | 0 | 2 | 0 | 2 | 1 | 0 | 0 | 7 |
| *TaXTH9-2D* | 1 | 0 | 0 | 1 | 0 | 2 | 1 | 2 | 0 | 1 | 0 | 8 |
| *TaXTH10-7A-1* | 3 | 0 | 1 | 0 | 0 | 3 | 2 | 6 | 0 | 0 | 0 | 15 |
| *TaXTH10-7A-2* | 2 | 0 | 0 | 2 | 1 | 1 | 3 | 4 | 0 | 1 | 0 | 14 |
| *TaXTH10-7A-3* | 0 | 1 | 0 | 0 | 1 | 7 | 2 | 0 | 0 | 1 | 1 | 13 |
| *TaXTH10-7A-4* | 0 | 2 | 1 | 0 | 3 | 2 | 1 | 2 | 0 | 0 | 0 | 11 |
| *TaXTH10-7A-5* | 1 | 0 | 0 | 0 | 0 | 3 | 0 | 0 | 2 | 3 | 1 | 10 |
| *TaXTH10-7B-1* | 2 | 0 | 1 | 0 | 0 | 4 | 2 | 18 | 2 | 0 | 0 | 29 |
| *TaXTH10-7B-2* | 0 | 0 | 0 | 0 | 1 | 3 | 0 | 0 | 0 | 0 | 0 | 4 |
| *TaXTH10-7B-3* | 1 | 0 | 1 | 1 | 0 | 1 | 2 | 0 | 0 | 2 | 0 | 8 |
| *TaXTH10-7D-1* | 2 | 0 | 1 | 0 | 0 | 2 | 1 | 0 | 0 | 1 | 0 | 7 |
| *TaXTH10-7D-2* | 0 | 0 | 0 | 0 | 1 | 3 | 0 | 4 | 0 | 0 | 0 | 8 |
| *TaXTH10-7D-3* | 0 | 0 | 1 | 3 | 0 | 2 | 1 | 2 | 0 | 3 | 0 | 12 |
| *TaXTH10-7D-4* | 0 | 0 | 0 | 0 | 0 | 6 | 1 | 8 | 0 | 1 | 0 | 16 |
| *TaXTH10-7D-5* | 0 | 0 | 0 | 2 | 2 | 6 | 2 | 6 | 0 | 2 | 0 | 20 |
| *TaXTH11-7A* | 0 | 0 | 1 | 0 | 0 | 7 | 1 | 4 | 2 | 1 | 0 | 16 |
| *TaXTH11-7B* | 0 | 1 | 1 | 1 | 0 | 11 | 2 | 6 | 2 | 1 | 0 | 25 |
| *TaXTH11-7D* | 1 | 1 | 1 | 1 | 0 | 6 | 3 | 6 | 4 | 1 | 0 | 24 |
| *TaXTH12-7A-1* | 2 | 0 | 1 | 0 | 0 | 6 | 2 | 6 | 0 | 1 | 0 | 18 |
| *TaXTH12-7A-2* | 1 | 0 | 2 | 1 | 0 | 5 | 1 | 4 | 0 | 0 | 0 | 14 |
| *TaXTH12-7B* | 0 | 0 | 1 | 1 | 0 | 3 | 3 | 6 | 1 | 0 | 0 | 15 |
| *TaXTH12-7D* | 0 | 0 | 0 | 0 | 0 | 2 | 3 | 2 | 0 | 2 | 0 | 9 |
| *TaXTH13/14-2A* | 3 | 0 | 0 | 0 | 1 | 6 | 0 | 0 | 0 | 1 | 0 | 11 |
| *TaXTH13/14-2B* | 0 | 0 | 0 | 0 | 2 | 5 | 1 | 6 | 0 | 1 | 0 | 15 |
| *TaXTH13/14-2D* | 0 | 0 | 1 | 1 | 0 | 6 | 1 | 4 | 1 | 2 | 0 | 16 |
| *TaXTH15-7A* | 1 | 1 | 0 | 3 | 0 | 4 | 1 | 6 | 1 | 1 | 0 | 18 |
| *TaXTH15-7B* | 0 | 1 | 0 | 0 | 0 | 2 | 0 | 6 | 0 | 5 | 0 | 14 |
| *TaXTH15-7D* | 1 | 1 | 1 | 2 | 1 | 2 | 3 | 4 | 1 | 3 | 0 | 19 |
| *TaXTH16-2A* | 3 | 0 | 1 | 0 | 0 | 9 | 4 | 6 | 0 | 3 | 0 | 26 |
| *TaXTH16-2B* | 0 | 0 | 0 | 1 | 0 | 6 | 3 | 6 | 0 | 4 | 0 | 20 |
| *TaXTH16-2D* | 0 | 0 | 1 | 0 | 0 | 9 | 0 | 8 | 0 | 1 | 0 | 19 |
| *TaXTH17-1A* | 0 | 0 | 0 | 0 | 0 | 0 | 0 | 4 | 0 | 3 | 0 | 7 |
| *TaXTH17-1B* | 0 | 0 | 0 | 2 | 0 | 1 | 2 | 2 | 0 | 2 | 0 | 9 |
| *TaXTH17-1D* | 0 | 0 | 0 | 1 | 0 | 3 | 0 | 4 | 0 | 0 | 0 | 8 |
| *TaXTH18-7A* | 0 | 0 | 0 | 0 | 0 | 4 | 1 | 6 | 3 | 2 | 0 | 16 |
| *TaXTH18-7B* | 0 | 0 | 0 | 1 | 0 | 10 | 1 | 4 | 3 | 0 | 0 | 19 |
| *TaXTH18-7D* | 0 | 0 | 1 | 0 | 0 | 4 | 2 | 6 | 3 | 0 | 0 | 16 |
| *TaXTH20/19-4B* | 2 | 0 | 1 | 0 | 0 | 0 | 3 | 6 | 0 | 1 | 0 | 13 |
| *TaXTH20/19-4D* | 2 | 0 | 0 | 2 | 2 | 2 | 2 | 8 | 0 | 1 | 0 | 19 |
| *TaXTH20/19-5A* | 1 | 0 | 0 | 2 | 0 | 1 | 1 | 6 | 0 | 1 | 0 | 12 |
| *TaXTH21-7A* | 1 | 0 | 0 | 0 | 0 | 1 | 0 | 8 | 0 | 0 | 0 | 10 |
| *TaXTH21-7B* | 0 | 0 | 1 | 2 | 0 | 1 | 0 | 6 | 0 | 0 | 0 | 10 |
| *TaXTH21-7D* | 1 | 0 | 1 | 1 | 0 | 5 | 0 | 10 | 0 | 1 | 0 | 19 |
| *TaXTH23-6A* | 2 | 0 | 0 | 1 | 0 | 3 | 0 | 6 | 0 | 1 | 0 | 13 |
| *TaXTH23-6B* | 0 | 0 | 1 | 0 | 0 | 6 | 0 | 4 | 2 | 2 | 0 | 15 |
| *TaXTH23-6D* | 2 | 0 | 2 | 1 | 0 | 2 | 0 | 4 | 1 | 2 | 0 | 14 |
| *TaXTH24-4A* | 1 | 0 | 1 | 0 | 1 | 1 | 2 | 2 | 0 | 1 | 1 | 10 |
| *TaXTH24-5B* | 0 | 0 | 1 | 0 | 0 | 2 | 1 | 2 | 0 | 0 | 0 | 6 |
| *TaXTH24-5D* | 0 | 0 | 1 | 1 | 0 | 2 | 3 | 2 | 0 | 1 | 0 | 10 |
| *TaXTH25-1A* | 0 | 0 | 0 | 0 | 2 | 3 | 0 | 6 | 0 | 0 | 0 | 11 |
| *TaXTH25-1B* | 2 | 0 | 1 | 2 | 1 | 1 | 1 | 4 | 0 | 2 | 0 | 14 |
| *TaXTH25-1D* | 1 | 0 | 0 | 0 | 1 | 0 | 1 | 2 | 0 | 2 | 0 | 7 |
| *TaXTH26* | 1 | 0 | 0 | 3 | 0 | 2 | 1 | 4 | 1 | 4 | 0 | 16 |
| *TaXTH26-6A* | 2 | 0 | 1 | 1 | 0 | 0 | 2 | 2 | 1 | 2 | 0 | 11 |
| *TaXTH26-6B* | 1 | 0 | 0 | 3 | 2 | 6 | 0 | 4 | 0 | 0 | 0 | 16 |
| *TaXTH28/27-4A* | 1 | 0 | 0 | 0 | 0 | 2 | 0 | 4 | 0 | 1 | 0 | 8 |
| *TaXTH28/27-4B* | 2 | 0 | 1 | 0 | 0 | 4 | 0 | 6 | 1 | 1 | 0 | 15 |
| *TaXTH28/27-4D* | 1 | 0 | 2 | 0 | 0 | 1 | 0 | 4 | 0 | 2 | 0 | 10 |
| *TaXTH29-5A* | 1 | 0 | 1 | 0 | 1 | 3 | 4 | 4 | 0 | 0 | 0 | 14 |
| *TaXTH29-5B* | 3 | 0 | 0 | 3 | 0 | 4 | 1 | 2 | 0 | 0 | 0 | 13 |
| *TaXTH29-5D* | 3 | 0 | 0 | 2 | 1 | 1 | 1 | 0 | 0 | 2 | 0 | 10 |
| *TaXTH30-6A* | 0 | 0 | 1 | 0 | 0 | 2 | 2 | 6 | 1 | 2 | 0 | 14 |
| *TaXTH30-6B* | 0 | 0 | 0 | 0 | 0 | 1 | 2 | 6 | 0 | 1 | 0 | 10 |
| *TaXTH30-6D* | 1 | 0 | 1 | 0 | 1 | 5 | 1 | 6 | 1 | 2 | 0 | 18 |
| *TaXTH31-3A-1* | 2 | 1 | 0 | 0 | 1 | 1 | 0 | 2 | 0 | 1 | 0 | 8 |
| *TaXTH31-3A-2* | 2 | 0 | 0 | 0 | 0 | 8 | 0 | 4 | 1 | 1 | 0 | 16 |
| *TaXTH31-3A-3* | 3 | 0 | 1 | 2 | 2 | 7 | 1 | 8 | 0 | 2 | 0 | 26 |
| *TaXTH31-3A-4* | 1 | 0 | 0 | 3 | 0 | 3 | 0 | 6 | 1 | 1 | 0 | 15 |
| *TaXTH31-3B-1* | 3 | 0 | 1 | 0 | 0 | 5 | 3 | 2 | 0 | 0 | 0 | 14 |
| *TaXTH31-3B-2* | 2 | 0 | 0 | 0 | 1 | 5 | 2 | 2 | 0 | 1 | 0 | 13 |
| *TaXTH31-3B-3* | 2 | 0 | 0 | 0 | 0 | 3 | 0 | 0 | 0 | 0 | 0 | 5 |
| *TaXTH31-3D-1* | 2 | 0 | 0 | 0 | 0 | 5 | 0 | 2 | 2 | 1 | 0 | 12 |
| *TaXTH31-3D-2* | 1 | 0 | 0 | 0 | 0 | 8 | 1 | 2 | 1 | 2 | 0 | 15 |
| *TaXTH31-3D-3* | 2 | 1 | 2 | 1 | 0 | 4 | 1 | 0 | 0 | 2 | 0 | 13 |
| *TaXTH31-3D-4* | 2 | 0 | 0 | 0 | 0 | 1 | 1 | 10 | 1 | 0 | 0 | 15 |
| *TaXTH32-4A-1* | 2 | 0 | 0 | 1 | 0 | 2 | 2 | 0 | 0 | 0 | 0 | 7 |
| *TaXTH32-4A-2* | 1 | 1 | 0 | 1 | 0 | 9 | 0 | 4 | 1 | 3 | 0 | 20 |
| *TaXTH32-7A-1* | 1 | 0 | 0 | 0 | 0 | 1 | 1 | 4 | 0 | 0 | 0 | 7 |
| *TaXTH32-7A-2* | 1 | 0 | 1 | 0 | 0 | 5 | 1 | 6 | 1 | 1 | 0 | 16 |
| *TaXTH32-7D-1* | 0 | 0 | 0 | 1 | 1 | 2 | 5 | 4 | 0 | 3 | 0 | 16 |
| *TaXTH32-7D-2* | 0 | 1 | 0 | 1 | 0 | 2 | 0 | 10 | 3 | 4 | 0 | 21 |
| *TaXTH33-5B* | 0 | 0 | 0 | 0 | 0 | 2 | 2 | 8 | 0 | 2 | 0 | 14 |
| *TaXTH33-5D* | 2 | 0 | 0 | 0 | 0 | 3 | 1 | 6 | 0 | 0 | 0 | 12 |
| *TaXTH33-7A* | 1 | 0 | 1 | 1 | 0 | 3 | 0 | 4 | 0 | 0 | 0 | 10 |
| *TaXTH33-7D* | 0 | 0 | 1 | 1 | 0 | 1 | 1 | 2 | 0 | 4 | 1 | 11 |
| *TaXTH34-2A-1* | 2 | 0 | 0 | 0 | 1 | 2 | 0 | 6 | 1 | 5 | 0 | 17 |
| *TaXTH34-2A-2* | 0 | 0 | 0 | 2 | 0 | 3 | 2 | 4 | 0 | 2 | 0 | 13 |
| *TaXTH34-2A-3* | 0 | 0 | 1 | 0 | 0 | 6 | 1 | 12 | 0 | 1 | 0 | 21 |
| *TaXTH34-2B* | 1 | 0 | 1 | 1 | 0 | 5 | 1 | 2 | 0 | 2 | 0 | 13 |
| *TaXTH34-2D-1* | 0 | 0 | 0 | 0 | 0 | 3 | 3 | 0 | 0 | 2 | 0 | 8 |
| *TaXTH34-2D-2* | 0 | 0 | 0 | 0 | 0 | 2 | 2 | 6 | 0 | 2 | 0 | 12 |
| *TaXTH34-2D-3* | 1 | 0 | 0 | 0 | 0 | 4 | 0 | 4 | 0 | 2 | 0 | 11 |
| *TaXTH34-2D-4* | 1 | 1 | 0 | 1 | 0 | 4 | 4 | 2 | 0 | 2 | 0 | 15 |
| *TaXTH35-3A* | 0 | 1 | 1 | 0 | 0 | 3 | 0 | 0 | 0 | 0 | 0 | 5 |
| *TaXTH35-3D* | 2 | 1 | 2 | 0 | 1 | 1 | 3 | 6 | 0 | 0 | 0 | 16 |
| *TaXTH36-3A* | 1 | 0 | 0 | 0 | 1 | 3 | 1 | 0 | 0 | 0 | 0 | 6 |
| *TaXTH36-3B* | 0 | 0 | 1 | 0 | 0 | 5 | 1 | 2 | 1 | 1 | 0 | 11 |
| *TaXTH36-3D* | 0 | 0 | 2 | 0 | 1 | 2 | 2 | 0 | 0 | 1 | 0 | 8 |
| *TaXTH37-3A* | 0 | 1 | 0 | 0 | 1 | 1 | 0 | 2 | 0 | 0 | 0 | 5 |
| *TaXTH37-3B-1* | 0 | 0 | 0 | 1 | 1 | 1 | 0 | 0 | 0 | 2 | 0 | 5 |
| *TaXTH37-3B-2* | 1 | 1 | 2 | 0 | 1 | 2 | 2 | 2 | 0 | 1 | 0 | 12 |
| *TaXTH37-3D-1* | 0 | 0 | 2 | 0 | 3 | 0 | 1 | 0 | 0 | 0 | 0 | 6 |
| *TaXTH37-3D-2* | 0 | 0 | 0 | 0 | 0 | 1 | 0 | 0 | 0 | 1 | 0 | 2 |
| *TaXTH38-7A* | 0 | 0 | 1 | 1 | 0 | 5 | 2 | 6 | 0 | 2 | 0 | 17 |
| *TaXTH38-7B* | 0 | 1 | 0 | 1 | 0 | 3 | 2 | 8 | 0 | 1 | 0 | 16 |
| *TaXTH38-7D-1* | 1 | 0 | 1 | 1 | 0 | 3 | 2 | 8 | 0 | 1 | 0 | 17 |
| *TaXTH38-7D-2* | 0 | 0 | 1 | 1 | 0 | 3 | 2 | 8 | 0 | 2 | 0 | 17 |
| *TaXTH38-7D-3* | 1 | 0 | 0 | 0 | 0 | 3 | 2 | 8 | 3 | 2 | 0 | 19 |
| *TaXTH38-7D-4* | 0 | 0 | 1 | 0 | 1 | 8 | 1 | 4 | 1 | 1 | 0 | 17 |
| *TaXTH39-7A* | 0 | 0 | 2 | 0 | 0 | 0 | 1 | 0 | 0 | 0 | 0 | 3 |
| *TaXTH39-7B* | 0 | 0 | 1 | 0 | 0 | 0 | 0 | 2 | 0 | 1 | 0 | 4 |
| Total number of genes | 71 | 18 | 61 | 56 | 34 | 126 | 96 | 112 | 42 | 98 | 5 |  |
|  |  |  |  |  |  |  |  |  |  |  |  |  |

**Table S3. Primers used in this study.**

| Purpose | Primer name | Primer sequence |
| --- | --- | --- |
| RT-qPCR | TaXTH17-1D-F | CGACTTCCACACCTACAAGATCG |
|  | TaXTH17-1D-R | CAGGTCGTCGTACTTCTTGAACG |
|  | TaGAPDH-F | AGTTCATGCCATGACTGCAA |
|  | TaGAPDH-R | CCAGTGCTGCTTGGAATGATG |
| Vector construction for ectopic expression in yeast | Y-TaXTH17-1D-F | CCGCCAGTGTGCTGGAATTCATGGCGAGGCCGTCC |
|  | Y-TaXTH17-1D-R | GATGGATATCTGCAGAATTCTCAGTTGCGGTTGCACTCGG |
| Vector construction for subcellular localization and overexpression in Arabidopsis | A-TaXTH17-1D-F | GGACAGCCCAGATCACTAGTATGGCGAGGCCGTCC |
|  | A-TaXTH17-1D-R | CCCTTGCTCACCATGGATCCGTTGCGGTTGCACTCGG |
| Vector construction for VIGS in wheat | TaXTH17-F | cttgtccttggagctgaagc |
|  | TaXTH17-R | gaggccgggttctacgac |
|  |  |  |
